# Enhanced Activity in Layered Metal-Oxide-Based Oxygen Evolution Catalysts by Layer-by-Layer Modulation of Metal-Ion Identity

**DOI:** 10.1021/acscatal.5c02788

**Published:** 2025-11-24

**Authors:** Ran Ding, Daniel Maldonado-Lopez, Jacob E. Henebry, Jose Mendoza-Cortes, Michael J. Zdilla

**Affiliations:** † Department of Chemistry, 6558 Temple University, 1901 N. 13th St., Philadelphia, Pennsylvania 19122, United States; ‡ Department of Chemical Engineering & Materials Science, 3078 Michigan State University, East Lansing, Michigan 48824, United States; § Department of Physics & Astronomy, 3078 Michigan State University, East Lansing, Michigan 48824, United States

**Keywords:** water oxidation, water splitting, layered materials, electrocatalysis, heterogeneous catalysis

## Abstract

Few-layered potassium nickel and cobalt oxides show drastic differences in catalytic activity based on metal ion preorganization. Uniform compositions [(CoO_2_/K)_6_ or (NiO_2_/K)_6_] show limited activity, while homogeneously mixed-metal cobalt/nickel oxides [(Co_*n*_Ni_(1–*n*)_O_2_/K)_6_] display moderate improvement. However, a layer-by-layer arrangement of alternating cobalt and nickel oxide sheets [e.g., (CoO_2_/K/NiO_2_/K)] provides superior catalytic performance, reducing the oxygen evolution overpotential by ∼200–400 mV. Density functional theory simulations provide an illustration of the electronic properties (density of states and localization of orbitals) that promote catalysis in the layer-segregated materials over those of homogeneous composition. This study reveals that atomic preorganization of metal ions within layered catalysts plays a more crucial role than the overall metal composition in enhancing catalytic efficiency for oxygen evolution.

## Graphical abstract

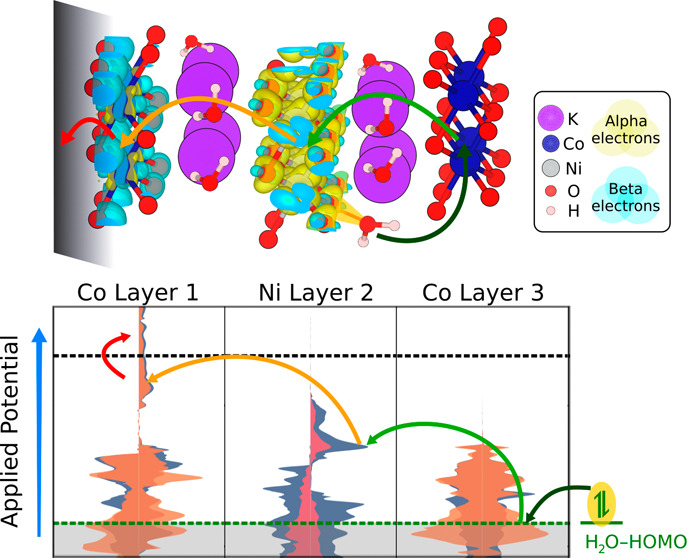

## Introduction

The pursuit of solar water splitting as an alternative to environmentally harmful burning of fossil fuels has led to extensive research on heterogeneous catalysts for water oxidation: the most challenging half of the water splitting reaction. The generation of reducing equivalents, protons, and O_2_ from water is challenging due to the stability of water and the common formation of reactive oxygen species as undesirable partially oxidized products.^1^ The development of catalysts that perform the full four-electron oxidation of two water molecules without deleterious reactive oxygen species formation is of central importance. Although iridium and ruthenium oxides have served as benchmark oxygen evolution reaction (OER) catalysts, their high cost limits their applicability. Recently, layered materials of first-row transition metals, including nickel and cobalt, have emerged as promising, cost-effective alternatives. However, most studies on these catalysts focus on metal composition rather than atomic-scale arrangement, leaving the effect of metal ion organization on the catalytic efficiency largely unexplored.

The layered double hydroxides are mixed-metal layered catalytic materials that constitute a particularly active class of catalysts that have received much recent attention for their ability to catalyze the OER at low overpotentials.^2^ Other related cobalt-containing mixed-metal oxides, hydroxides, and alkoxides^3^ have also shown excellent catalytic properties. Studies on layered nickel and cobalt oxides also demonstrate promise for these metal oxides in water oxidation.^4,5^ Works from our group and others have explored the layered manganese oxide birnessite in great detail and uncovered details about the role of interior cation identity,^6–11^ oxidation state,^8,12,13^ solvent frustration,^7,14,15^ and defect density^12,13^ on the activity of these catalysts. Important for this study is our finding that due to the layered nature of these catalysts, overpotentials are influenced by the charge transfer resistance (as measured by electrochemical impedance spectroscopy), but that Tafel slopes are not affected, suggesting the interlayer resistivity is unchanged, as is the kinetic barrier in homogeneously layered systems.^15^ However, our interest has turned to mixed-metal systems, as we found that doping of other transition metals into birnessite^10,11,16^ has given rise to the most substantial improvements in catalysis. The use of dopants to introduce defect sites is a well-known strategy in OER catalysis; however, the approach normally presumes a chemical role played by the dopant, where overpotentials may be lowered by interface restructuring and tuning.^17^

While birnessite traditionally requires a very large overpotential of 700 mV or more to catalyze the OER (at 10 mA), simple protocols to include defects and dopants in birnessite have brought the potential to less than 400 mV, making these modified birnessites superior to any class of pure manganese-based heterogeneous OER catalysts. The use of elemental mixtures in heterogeneous catalysis in general has been a successful approach to improving OER activity,^6–8,11^ primarily due to alterations in the density of states (DOS) imposed by the dopant orbitals, which can improve hole migration, substrate binding, and transition state stabilization. However, in most studies, the dopants are distributed randomly throughout the structure, with little attention paid to how the organization of heterometallic structures affects catalysis.

A recent study from one of our groups demonstrated a remarkable dependence of the OER on the distribution of catalytically important Mn^III^ in few-layer birnessites. Birnessite is a hydrated, layered manganese oxide with the general formula K_*x*_MnO_2_(H_2_O)_*y*_. The MnO_2_ atomically thin layers are mostly Mn^IV^-based but contain some amount (typically less than half) of Mn^III^. These Mn^III^ centers impart a negative charge to the sheet, which is balanced by hydrated interlayer potassium ions. While Mn^III^ has long been known to promote OER in manganese-based catalysts,^18^ a theoretical study from Perdew and collaborators predicted that Mn^III^ abundance was not the main key to catalytic activity in birnessite but rather the organization of Mn^III^-rich layers proximal to Mn^III^-poor layers. These “potential steps” between electron-rich and electron-poor sheets were predicted to facilitate electron transport across layers by positioning the small polaronic e_g_^1^ state at the valence band maximum of the Mn^III^-rich layer at a similar energy to the conduction band minimum of the adjacent Mn^III^-poor layer, resulting in enhanced electron transfer. We showed in this work that few-layer catalysts with such alternating layers of Mn^III^-rich and -poor layers were superior catalysts to systems constructed entirely from Mn^III^-rich layers.^13^ Furthermore, we confirmed the theoretically predicted changes to the DOS using scanning tunneling spectroscopy, which showed the expected repositioning of band edges of bilayers of Mn^III^-rich and -poor sheets.^12^

Since DOS is dependent not only upon the oxidation state of metals but also (and even more so) on metal identity, we report here how modulation of DOS in layered materials by a more obvious approach—the alternation of metal identity in neighboring layers—would affect the OER activity. We report the preparation of heterostructured few-layer nickel and cobalt oxide materials with interstitial hydrated potassium ions. This is achieved by the exfoliation of pure phases of the layered material, followed by a layer-by-layer reassembly onto fluorine-doped tin oxide (FTO) electrodes with interlayer hydrated potassium ions, making these materials analogous to potassium birnessite but with layers of pure cobalt oxide (Co) or nickel oxide (Ni), or heterostructures of each. The layer-by-layer reassembly protocol permits exquisite control of the layer order, permitting an examination of the role of the position and number of potential steps in these structures. The result shows that the heterostructures facilitate catalysis and that the activity of these ordered materials is greater than the sum of their parts.

## Results and Discussion

### Synthesis and Few-Layer Catalyst Assembly

LiNiO_2_ and LiCoO_2_ were prepared using published protocols.^4,19^ For control experiments, we also prepared LiCo_*x*_Ni_(1–*x*)_O_2_ samples (*x* = 1/3, 1/2, 2/3) with homogeneously distributed cobalt and nickel within the sheets (i.e., solid solutions).

The layer morphology is confirmed by transmission electron microscopy (TEM) (Figures S1–S3). All samples share a similar structure, as characterized by powder X-ray diffraction (PXRD) (Figure 1). The position of the (003) peak around 2θ = 19° indicates that LiNiO_2_ has a larger lattice parameter *c* than LiCoO_2_, which is consistent with the literature.^20^ The elemental composition was confirmed by inductively coupled plasma-optical emission spectroscopy (ICP-OES).

**Figure 1:**
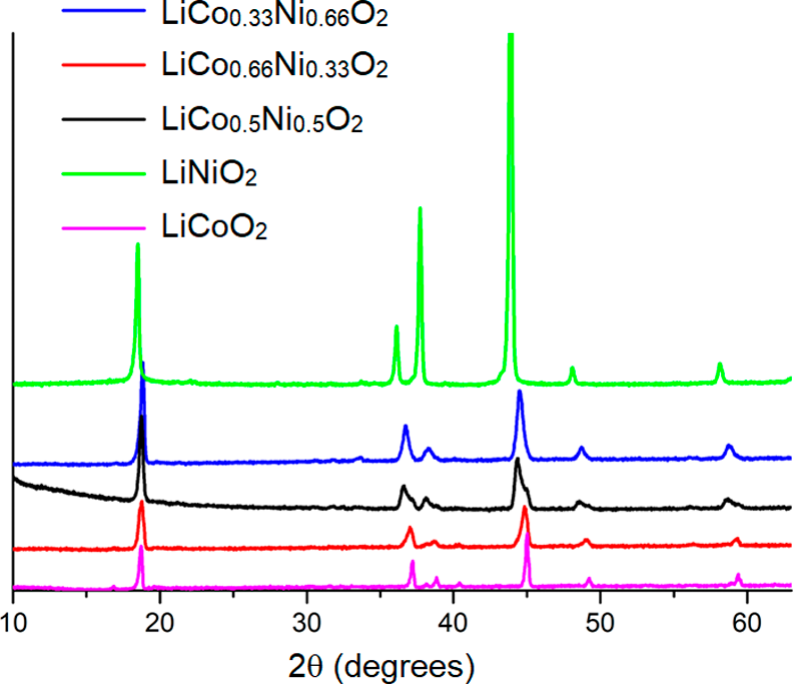
PXRD patterns of synthetic LiCo_*x*_Ni_1–*x*_O_2_ with *x* = 0, 0.33, 0.50, 0.66, and 1. The 003 peak at 2θ = 19° corresponds to the interlayer spacing. The region between 2θ = 37–40 comprises three reflections (101, 006, and 10), which have slightly different intensities stemming from changes in atomic composition. The positions exhibit a gradual shift to higher 2θ, as the cobalt content increases due to the slightly smaller lattice constants of LiCoO_2_.^22^

Using an exfoliation and reassembly approach,^12,15,21^ we precisely controlled MO_2_ stacking to produce few-layered catalysts with systematic layer organization. First, we exfoliated the metal oxides into single-layer nanosheets (NSs) of stoichiometric [Co^III^O_2_]_*n*_ *^n-^* and [Ni^III^O_2_]_*n*_^*n*–^ via insertion of bulky tetrabutylammonium (NBu_4_^+^) ions into the interlayer.^15^ The layered structure is maintained following exfoliation, as evidenced by TEM (Figures S1–S3).

A layer of polyethylenimine (PEI) was deposited on an FTO substrate to create a positively charged surface to which the first negative metal oxide sheet adheres. Subsequent sheets are excluded due to repulsion between the negatively charged sheets. The substrate is then rinsed and dipped in a potassium hydroxide solution to deposit a layer of potassium, which charge-balances the anionic NS. The substrate is then repeatedly coated in NS, followed by a rinse and then with K^+^ using a solution of KOH, etc. Repetition of this process results in stacking sequential layers of metal oxides with intervening layers of aqueous K^+^, to reassemble few-layer KMO_2_ with controllable structures (Figure 2). AFM results have shown that the expected interlayer spacing is re-established upon restacking of metal oxide sheets using this protocol. Furthermore, the deposition of a single layer per dip is supported by the gradual increase in optical absorption and the linear increase in charge-transfer resistance with each dip, consistent with the deposition of one layer per dip.^15^ Following completion of the coating process, the expected elemental composition is confirmed by XPS (vide infra).

**Figure 2:**
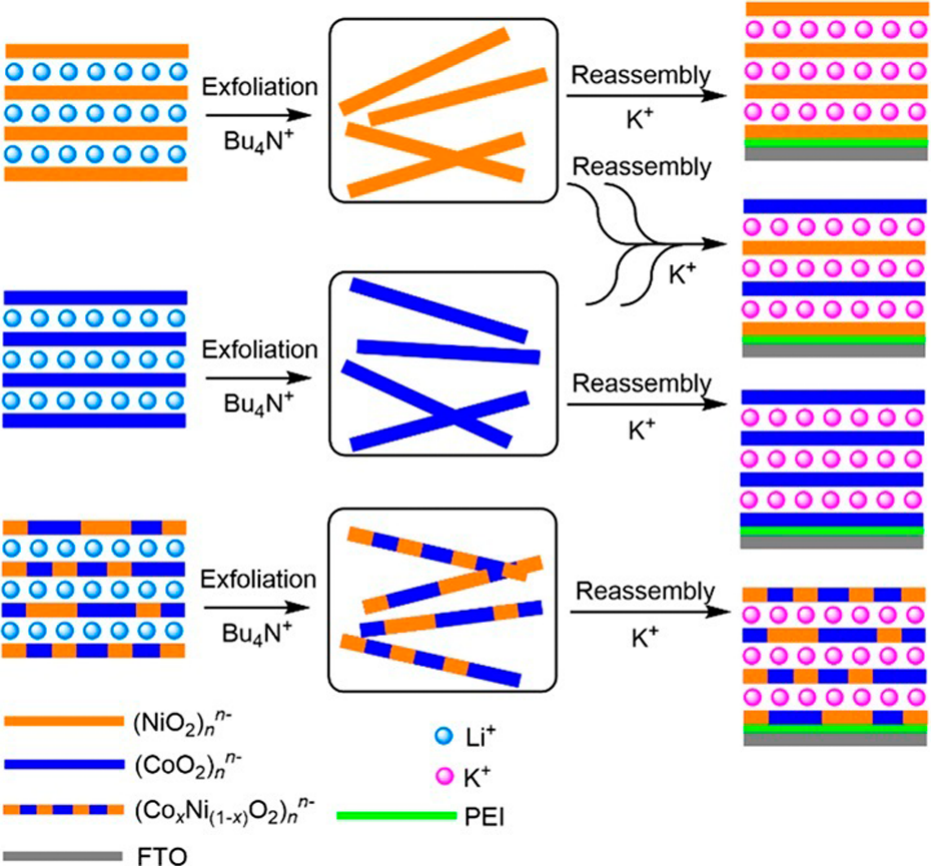
Schematic of the exfoliation and layer-by-layer reassembly process used to deposit metal oxide NS on PEI-coated FTO substrates. Successive coatings of metal oxide layers and intercalated potassium ions yield few-layer KMO_2_ structures with a precise layer order, enabling control over the atomic arrangement of Co and Ni oxide sheets.

Catalyst layer structure is denoted using, for example, (M/K)_6_ (M = Co, Ni), indicating six layers of metal oxide with six layers of potassium ions; the first layer is deposited onto PEI-coated FTO. For mixed-layer materials, the leftmost indicated metal is the one stacked first against FTO. Samples were prepared by six sequential coating cycles. (Co/K/Ni/K)_3_ was synthesized by stacking pure cobalt oxide layers with pure nickel oxide layers alternatively three times each, with K^+^ as intercalated cations. The few-layer material (Co_0.5_Ni_0.5_/K)_6_, as another example, was prepared by repeatedly stacking one layer of mixed/doped [Co_0.5_Ni_0.5_O_2_]^−^ with another layer of K^+^ six times. While it is possible for some catalyst restructuring and changes in defect density and activity of NS during the exfoliation and reassembly process, and during initial linear sweep voltammetry (LSV) sweeps, all samples, including all-nickel or all-cobalt control samples, are exfoliated and reassembled so that this variability is controlled for. The elemental composition and the fidelity of the stacking process are confirmed by 2p Co and Ni XPS analyses, which show the corresponding signals for cobalt and/or nickel depending on which layers have been added.

### Electrocatalysis

Water oxidation was carried out in 1 M KOH and corrected to the reversible hydrogen electrode (RHE). For [Co_0.5_Ni_0.5_O_2_]^−^, the overpotential at the current density of 5 mA/cm^2^ is ∼650 mV. As shown in Figure 3, the homogeneously alloyed structure (Co_0.5_Ni_0.5_/K)_6_ exhibits improved catalytic activity compared to pure cobalt oxide (Co/K)_6_ and pure nickel oxide (Ni/K)_6_. Notably, the layer-by-layer arrangement (Co/K/Ni/K)_3_, with alternating Co and Ni oxide layers, achieves an exceptional OER overpotential reduction to ∼ 460 mV, underscoring the impact of atomic-scale layer modulation on catalytic performance. Considering that both (Co_0.5_Ni_0.5_/K)_6_ and (Co/K/Ni/K)_3_ possess the same Co/Ni ratio (1:1) and the same metal oxide layer number, the improvement of the catalytic performance is attributed to the unique organized structure of the latter material. This structural feature alters the electronic structure of the material and thus improves its catalytic activity.

**Figure 3:**
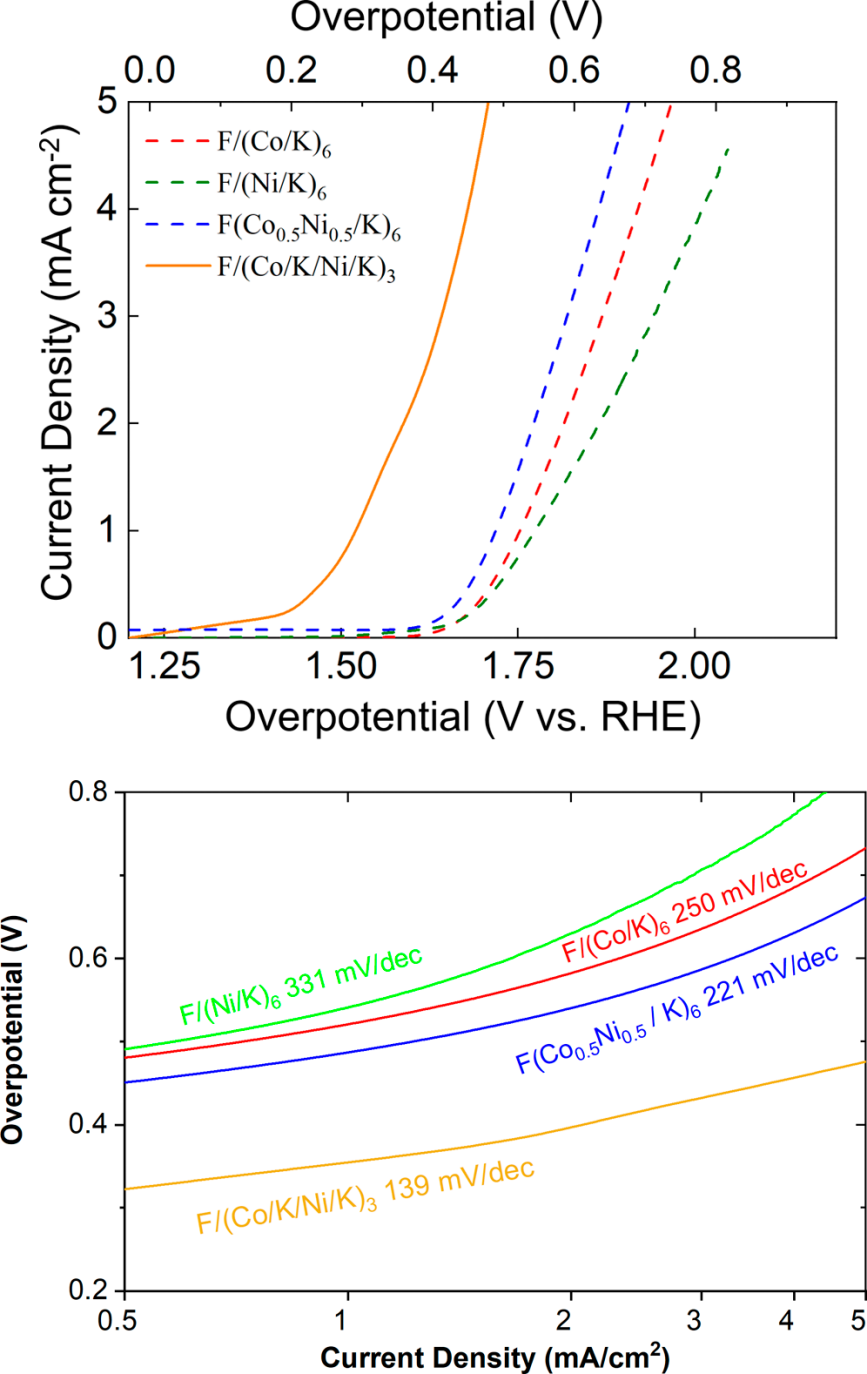
Top: LSV of few-layer materials (Co/K)_6_, (Ni/K)_6_, (Co_0.5_Ni_0.5_/K)_6_, and (Co/K/Ni/K)_3_. All samples are made by six metal oxide layers, and “F” represents FTO glass. Bottom: Tafel slopes derived from the LSV curves in the top.

In addition to the 1:1 Co/Ni ratio experiment discussed above, we have also applied this strategy to mixed-metal oxides with 1:2 metal ratios. As shown in Figure 4, when the ratio of Co to Ni is 1:2, (Co/K/Ni/K/Ni/K)_2_ gives an overpotential of ∼500 mV at a current density of 10 mA/cm^2^, while the overpotential of homogeneous (Co_0.33_Ni_0.66_/K)_6_ is much higher: ∼740 mV. When the Co/Ni ratio is 2:1 in (Ni/K/Co/K/Co/K)_2_, the material shows the best catalytic performance and the overpotential is ∼370 mV. By contrast, homogeneous (Co_0.66_Ni_0.33_/K)_6_ gives a much higher overpotential of ∼600 mV. Despite some expected variability sample-to-sample, these trends are robust across multiple trials and multiple samples (Figures 5 and S4). In Figure 5, we summarize the overpotential results with standard deviation bars. We can clearly see that mixed-metal oxides with alternatively distributed cobalt and nickel (circles) perform better than corresponding samples with a homogeneous distribution (squares). The chronoamperometry curves shown in Figure S5 demonstrate the alternatively stacked sample could be kept active for more than 1 h at an overpotential of less than 370 mV.

**Figure 4:**
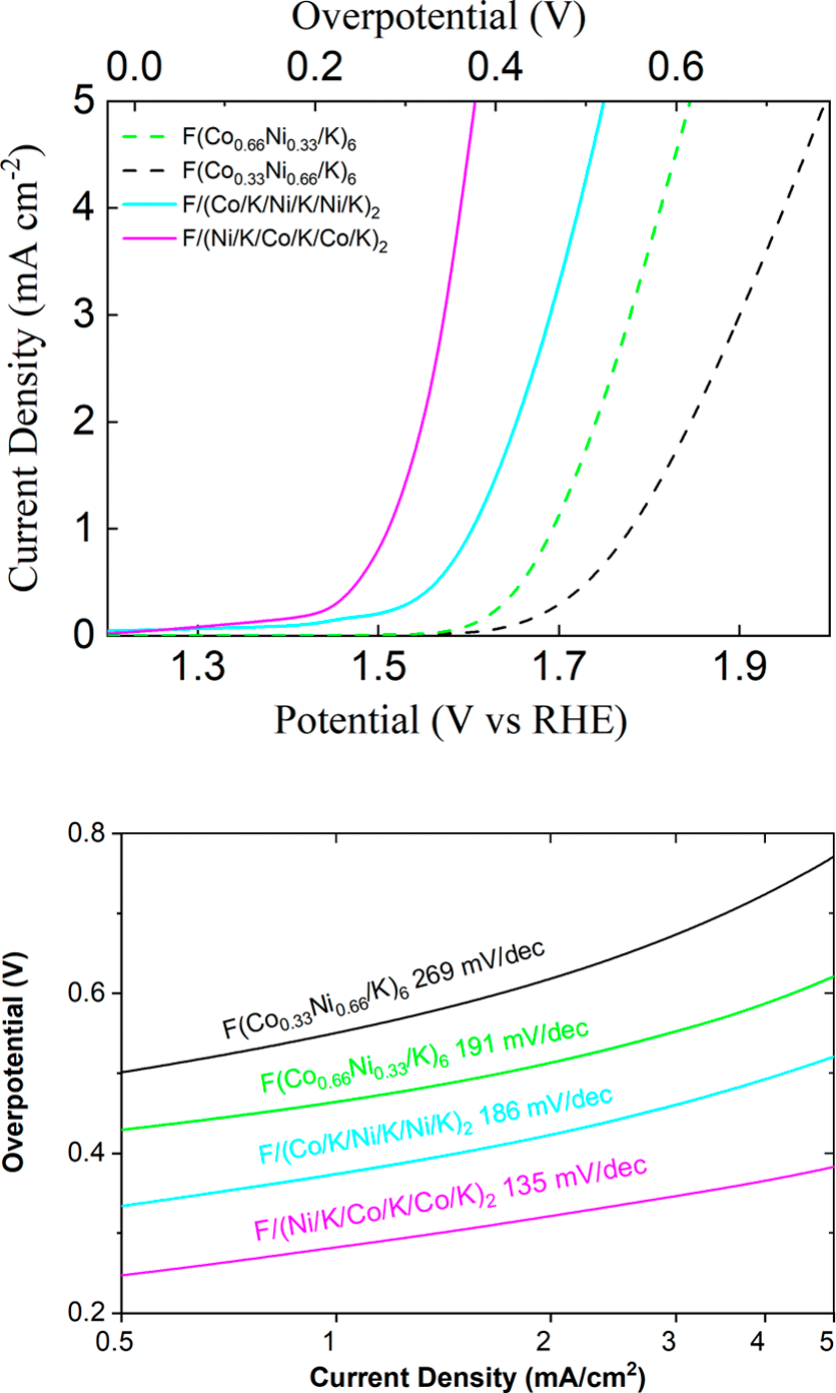
Top: LSV of few-layer materials (Co/K/Ni/K/Ni/K)_2_, (Ni/K/Co/K/Co/K)_2_, (Co_0.33_Ni_0.66_/K)_6_, and (Co_0.66_Ni_0.33_/K)_6_. All samples are made by six metal oxide layers, and “F” represents FTO glass. Bottom: Tafel slopes derived from the LSV curves in the top.

**Figure 5:**
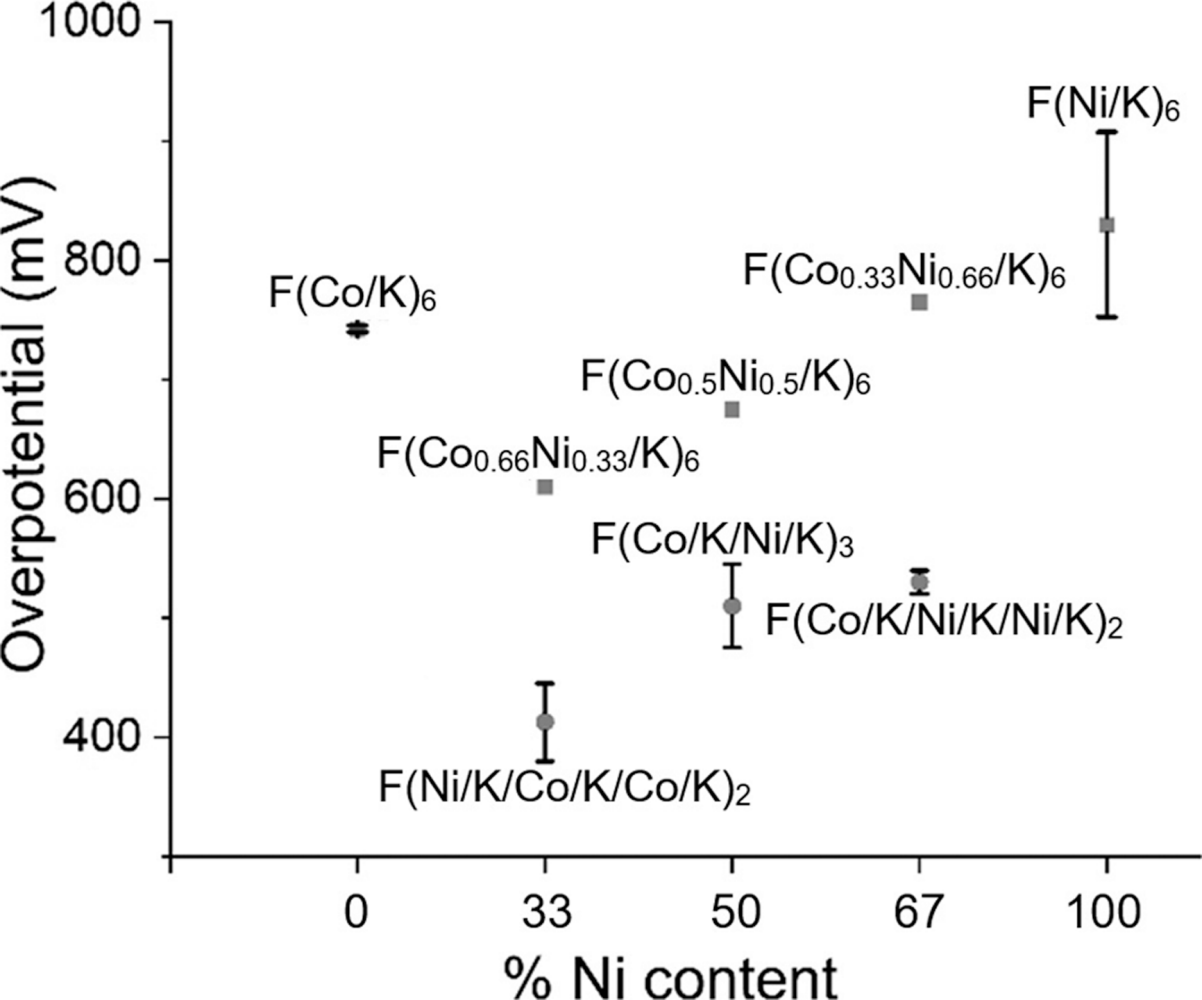
Overpotential of few-layer materials at 5 mA/cm^2^ with error bars.

Tafel analysis (Figures 3 and 4) reveals some noteworthy trends across these materials. The Tafel slope is influenced by kinetic barriers (in this case, we expect these to be electron transport barriers), while the vertical position is related to the number of charge carriers. While these values may be dependent on other impedance factors (such as mass transport, electrode surface area),^23^ these latter properties are well controlled in our use of analogous electrodes, surface area normalization, and the use of chemically analogous catalysts. Thus, the differences are consistent with the predicted change in the internal electron transport barriers. Across these materials generally, as the overpotential increases, the Tafel slope also increases, suggesting changes in activation energy are responsible for catalytic activity. The only exception is between the F/(Co/K/Ni/K/Ni/K)_2_ and the F/(Co_0.66_Ni_0.33_/K)_6_ samples (Figure 4), which have similar Tafel slopes, but different vertical positions. The lower vertical position of the modulated sample F/(Co/K/Ni/K/Ni/K)_2_ is consistent with the general observation that the modulation of DOS in adjacent layers provides more facile transport of electrons across the layers. While our results are consistent with a change in internal electron transfer rates, we cannot rule out the possibility of chemical cooperativity between nickel and cobalt, which could also result in reduced chemical activation barriers (in addition to electron transfer barriers) in the mixed-metal catalysts.

Elemental analysis (XPS) following electrocatalysis confirms that the atomic composition is mostly retained during and after the OER, though some signal loss is observed upon catalyst death. The XPS of the most active mixed material shows that during catalysis, cobalt is more prone to leaching than Ni, as the Co signal has decreased by about 50% postmortem (Figure S7A), while the Ni signals have remained at the same intensity (Figure S7B). In few-layered catalysts of nickel- or cobalt-only layers, the ions appear to leach equivalently (about 40% postmortem). With such thin-layered materials, structural analysis of the nanolayered substrate on FTO following electrocatalysis was not practical. However, past work indicates that layered LiNiO_2_ ^24^ and LiCoO_2_ ^25^ do not restructure under OER conditions, but delaminate and delithiate. In addition, the poorer performance of the mixed-composition sheets, e.g., F/(Co_0.5_Ni_0.5_/K)_6_, suggests that migration of transition metal ions across sheets to create in-layer mixtures is not responsible for improved catalysis. Even if structural rearrangement does occur during catalysis, the results show that the periodic modulation of metal-ion identity nevertheless improves catalytic performance.

While it is established from these experiments that alternating Co/K/Ni/K layers provide the best overpotentials, the question of whether the identity of the initial layer is important is of interest. We prepared a separate batch of catalysts wherein we compared six-layer alternating systems with nickel first, i.e., F/(Ni/K/Co/K)_6_, to those with cobalt first, F/(Co/K/Ni/K)_6_, and compared these to the pure phases F/(Co/K)_6_ and F/(Ni/K)_6_. In all cases, the compositionally modulated heterolayered materials were superior to the compositionally uniform materials, but we found that when the ratio of Co and Ni is unity, the stacking of cobalt layers first in F/(Co/K/Ni/K) improved the overpotential by 100 mV more than when nickel was stacked first in F/(Ni/K/Co/K)_6_, though stacking of nickel first in a mixed catalyst was still better than the all-nickel catalyst (see Figure S6). However, for mixtures of the metals in a 2:1 ratio, it was better to have 2:1 Co/Ni, even if Ni is layered first (Figure 4).

### Computational Studies

We used first-principles unrestricted hybrid density functional theory with Grimme-D3 dispersion corrections (HSE06-UDFT-D3 or DFT for simplicity) to compare the electronic properties of mixed-metal oxide vs pure metal oxide heterostructures. The HSE06 hybrid functional was chosen due to its effectiveness in accurately modeling electronic properties in layered oxides, and the Grimme-D3 dispersion correction addresses interlayer interactions critical to replicating the experimental conditions of layer-stacked materials. In Figure 6, we show the total band structures and DOS of the optimized bulk materials. We find that different-layer (Co/K/Ni/K)_∞_ and same-layer (Co_0.5_Ni_0.5_/K)_∞_ both present intermediate energy levels in the 1–2.5 eV range (coming mainly from Ni contributions) and in the 4.5–6 eV range (coming mainly from Co contributions), which are not present in the pure layered materials. The formation of intergap states is an excellent preliminary indication that K-intercalated mixed-metal oxide heterostructures allow better electron conduction throughout the structure (Figure 6).

**Figure 6:**
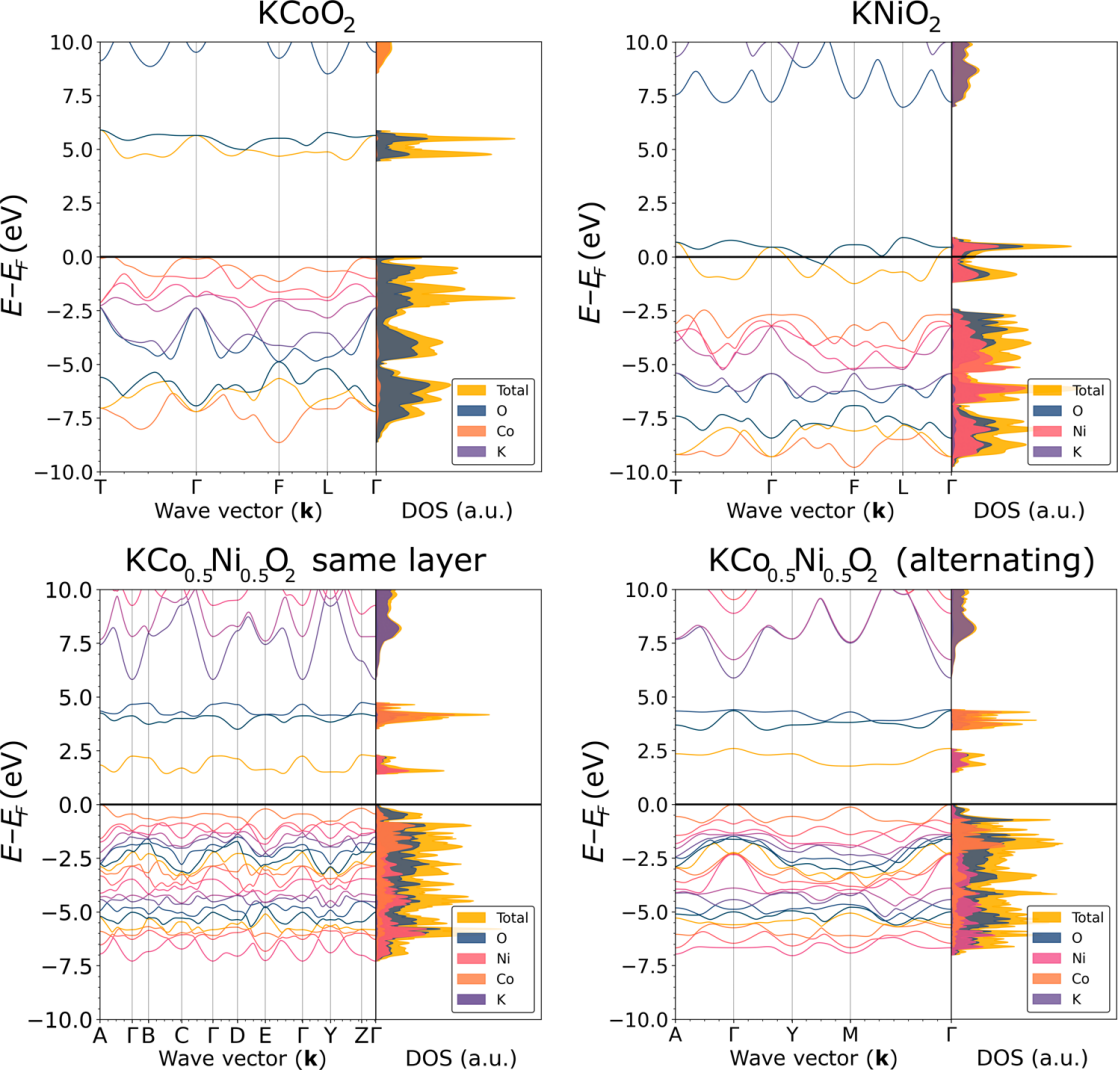
Band structures and total DOS for bulk KCoO_2_, KNiO_2_, same-layer KCo_0.5_Ni_0.5_O_2_, and different-layer KCo_0.5_Ni_0.5_O_2_ birnessites. The *y* axis is centered at the Fermi level.

Due to these intermediate energy levels, the bulk materials show promising behavior for increased catalytic activity in the mixed-transition-metal structures compared to the pure compounds. However, the bulk electronic properties do not replicate the trends observed in the experimental overpotentials of the few-layer materials. Moreover, the gaps in these bulk materials are too large to allow efficient electron conduction. Therefore, we next generated and analyzed the few-layer heterostructures. To accomplish this, we generated slabs, emulating experimental synthetic structures. These structures are periodic in the *x* and *y* directions, simulating infinitely large sheets. The KMO_2_ formula unit is repeated six times in the *z* direction. In Figure 7, we present the spin-polarized DOS for these few-layer compounds; in this figure, spin-up electrons are plotted to the right side and spin-down electrons to the left for each DOS plot. From the plots in Figure 7, it is evident that few-layered structures have better electron-transfer properties than bulk structures, as the gaps observed in bulk DOS close for almost all slab compounds, allowing for better conduction throughout the structure.

**Figure 7:**
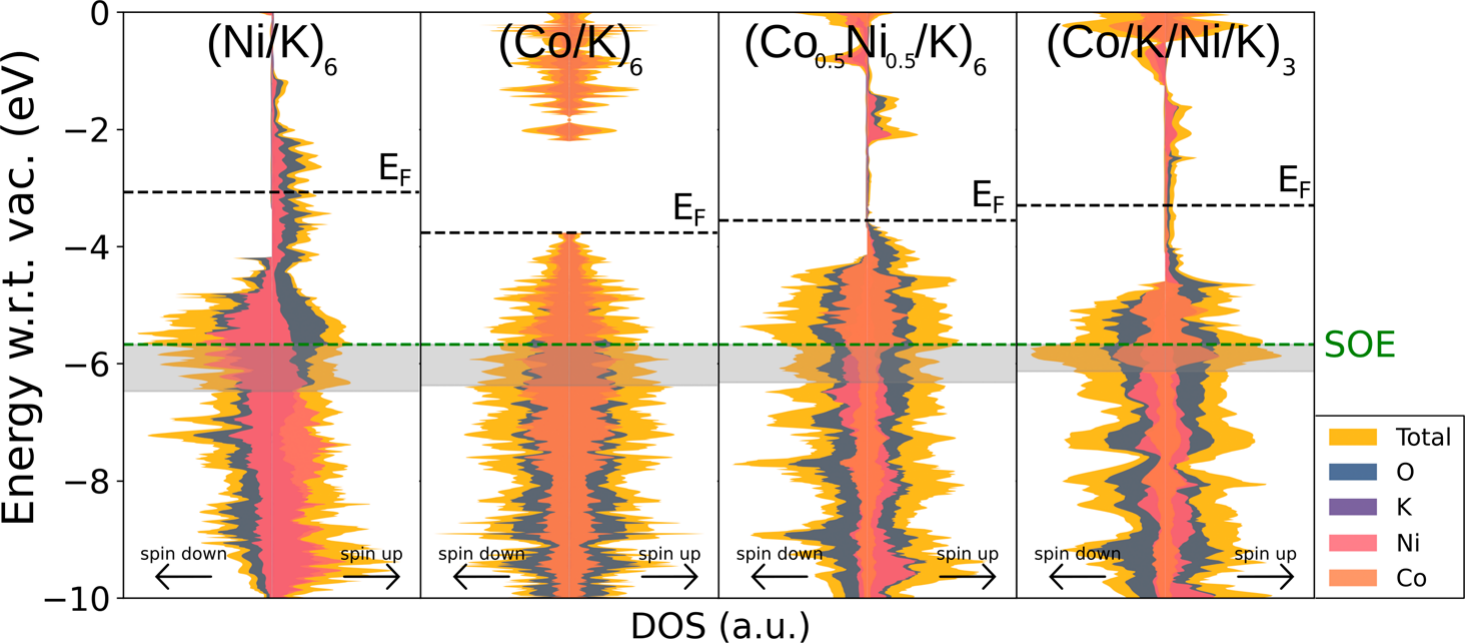
Electronic DOS of the optimized potassium-intercalated transition metal slabs. Contributions from each atomic species are included. Each plot was normalized with respect to its maximum peak in the −10 to 0 eV energy range (with respect to vacuum). The dotted green line indicates the potential for oxygen evolution to occur (SOE level, −5.67 eV with respect to vacuum). The filled rectangles represent the “SOE plus experimental overpotential” range. The dotted black line indicates the Fermi level. Spin-up electrons are plotted to the right, and spin-down electrons are to the left of each DOS plot.

Water splitting is thermodynamically possible at a voltage of 1.23 V vs SHE.^26^ This energy corresponds to −5.67 eV with respect to vacuum and is abbreviated the standard oxygen electrode (SOE) level. Therefore, it is important that catalysts for oxygen evolution provide enough electronic states at the SOE level, facilitating the generation of charge carriers with sufficient energy to participate in the reaction. This can be analyzed through the DOS plots in Figure 7. It is important to note that the plots were aligned with respect to vacuum to make direct comparisons between structures’ DOS profiles. Furthermore, the width of the DOS (*x* axis) was normalized to the largest DOS peak of each individual structure in the −10 to 0 eV range. In these plots, the dotted black line indicates the Fermi level and the dotted green line represents the SOE level (−5.67 eV with respect to vacuum). Furthermore, the filled gray rectangles indicate the SOE energy level plus the experimental overpotential (SOE+OP) of each corresponding structure (Figure 3). The SOE+OP region is an important descriptor of these materials’ catalytic performance as it indicates the electronic states that are likely being utilized during oxygen evolution catalysis.

The plots in Figure 7 are ordered from highest to lowest overpotentials (i.e., from worst to best water oxidation properties). It can be observed that (Ni/K)_6_ and (Co/K)_6_ present similar DOS profiles in the SOE+OP region. However, (Ni/K)_6_ presents an uneven distribution of α (spin-up) and β (spin-down) electrons with a smaller amount of spin-up states. This effect does not occur in (Co/K)_6_, which presents a symmetric distribution of α and β electronic states, likely contributing more total states around the critical SOE energy level, providing more reactive carriers, and resulting in a lower overpotential compared to the nickel slab. On the other hand, the mixed-composition-layer (Co_0.5_Ni_0.5_/K)_6_ heterostructure presents a higher α-electron peak at the SOE level and a relatively symmetric α and β electron distribution. Furthermore, all gaps close completely in this structure, resulting in better electron transport than the pure metal oxide slabs. Finally, the alternately stacked (Co/K/Ni/K)_3_ heterostructure presents the largest DOS peaks for both alpha and beta electrons exactly at the SOE+OP region, resulting in the highest number of available OER-catalytic states, which experimentally corresponds to the lowest overpotential of all calculated structures. Through this analysis, we observe a direct correspondence between the energy levels near the SOE level and the experimental overpotential. We believe that a large DOS at or slightly below the SOE could serve as a design principle to find low-overpotential oxygen-evolution electrocatalysts.

To further explore the catalytic properties in the alternately stacked heterostructure, we plot the layer-resolved DOS, as shown in Figure 8. Here, we observe that the outermost layers provide electron density around the Fermi level, in the −4 to −2 eV energy range. The appearance of these surface states is crucial for electron conduction in the structure, since the inner layers do not close the material’s band gap completely. Through this mechanism, electron conduction is facilitated at the surface. On the other hand, inner NiO_2_ layers provide states directly above and below the −4 to −2 eV range, facilitating electron conduction for different energy ranges. Oxygen atoms in the NiO_2_ layers make an important contribution to the DOS in the SOE+OP range (vide infra). Finally, inner CoO_2_ layers have strong contributions to the electron density in the SOE+OP range, especially stemming from Co atoms.

**Figure 8:**
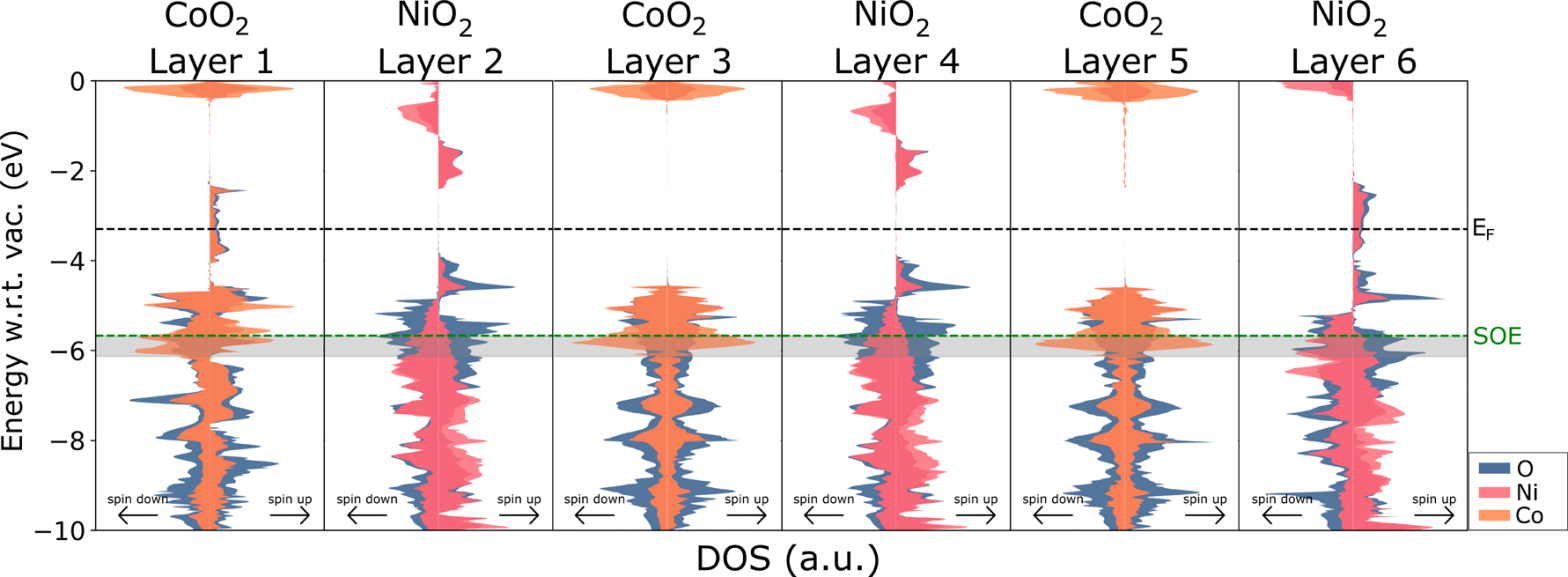
Layer-resolved DOS for the alternately stacked (Co/K/Ni/K)_3_ heterostructure. Layers are labeled 1–6, starting with CoO_2_ which is completely exposed to a vacuum and ending in NiO_2_ which has a potassium layer before the vacuum. The plots were normalized to 1/5 of the maximum total DOS peak in the −10 to 0 eV range. The dotted green line indicates the potential for oxygen evolution to occur (SOE level, or −5.67 eV with respect to vacuum). The filled rectangles represent the “SOE plus experimental overpotential” range. The dotted black line indicates the Fermi level. Spin-up electrons are plotted to the right and spin-down electrons to the left of each DOS plot.

As previously mentioned, oxygen in (Co/K/Ni/K)_3_ is one of the main contributors to electron density in the SOE+OP region. This effect does not occur as drastically in the pure metal oxide structures, indicating that oxygen atoms are likely important contributors to the enhanced catalytic properties of the alternately stacked heterostructure. To investigate this effect, we plot the spin density of the alternately stacked structure, as shown in Figure 9a. The spin density plot reveals a redistribution of electrons in the (Co/K/Ni/K)_3_ slab through the presence of pure nickel oxide layers separated by cobalt oxide layers. In particular, we observe that the oxygens directly bonded to nickel atoms present an open-shell triplet character. This triplet character can be observed in Figure 9b, which is a close-up of the outermost NiO_**2**_ layer. Triplet states are generally unstable for covalent bonds, but they are highly energetic and have been linked to catalytic activity during photosynthesis,^26^ and in transition metal catalysts.^27^ This redistribution of electron density is expected to promote the generation of reactive oxygen species, which likely serve as catalytic sites. Finally, in Figure 9c, we plot the orbital-resolved DOS for the alternately stacked (Co/K/Ni/K)_3_ heterostructure. Here, we observe that the main contributions to the DOS come from oxygen p-orbitals, cobalt d-orbitals, and nickel d-orbitals, with oxygen providing most of the electron density in the −10 to 0 eV range.

**Figure 9:**
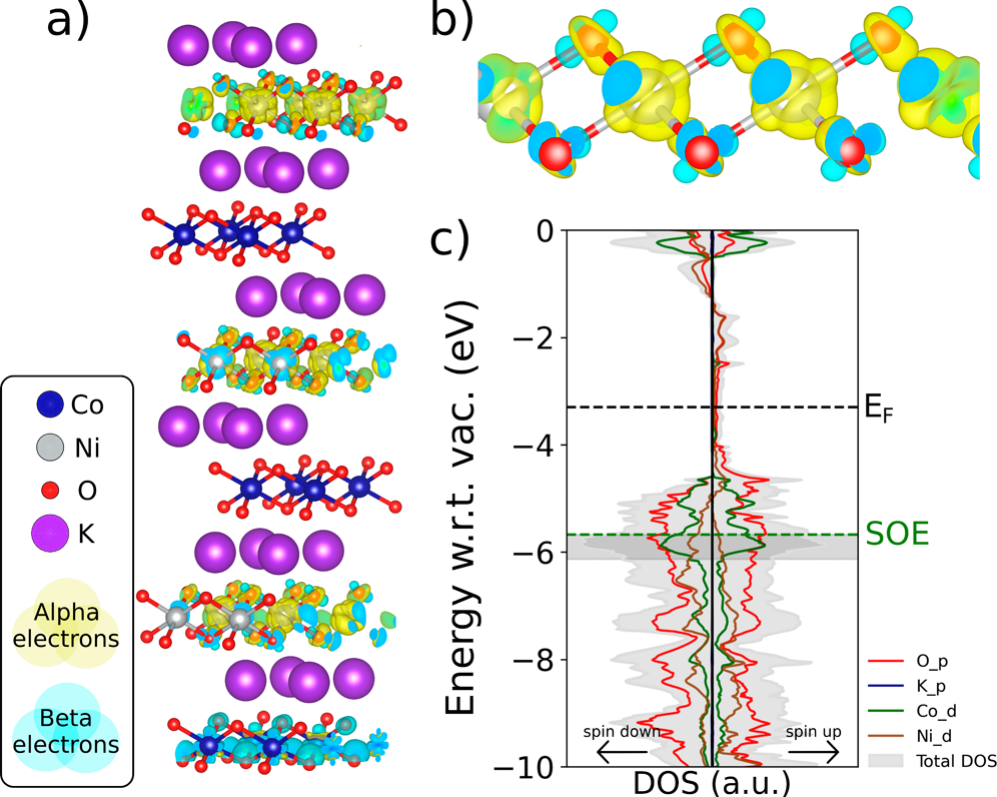
(Co/K/Ni/K)_3_ heterostructure electronic properties. (a) Spin density plot for the entire structure. (b) Spin density plot for the outermost NiO_2_ layer. (c) Orbital-resolved DOS. The dotted black line indicates the Fermi level; the dotted green line indicates the SOE level; and the filled rectangle represents the “SOE plus experimental overpotential” range. Spin-up electrons are plotted to the right and spin-down electrons to the left of the DOS plot.

In summary, calculations indicate that slab heterostructures present more prominent gap states than bulk materials and suggest that few-layer samples may experience a greater enhancement from these effects than bulk materials. Of the four calculated slabs, we find that the alternately stacked (Co/K/Ni/K)_3_ structure presents the best properties for oxygen evolution, since it provides (1) the largest amount of states at the SOE+OP energy range, (2) gap closure, providing better electron transport properties, (3) crucial states around the Fermi level localized at the surface of the slab, facilitating electron transport to the center of the slab, (4) inner-layer states spanning a wide range of energy levels, especially near the SOE level, and (5) oxygen atoms in NiO_2_ layers presenting an open-shell triplet character, expected to promote OER activity.

## Conclusions

This study reveals that controlling the atomic-scale distribution of metal ions within layered transition metal oxides is a more effective approach to enhancing the OER activity than altering the metal composition alone. Specifically, stacking discrete Co and Ni oxide layers rather than forming homogeneously mixed layers reduces the OER overpotential significantly. The superiority of preorganized structures with segregated metal identity in adjacent layers over solid solutions demonstrates that atomic organization is more important to catalytic activity than composition; samples with Co and Ni stacked separately possess better OER performance than those with Co and Ni homogeneously distributed within the layers. The improvement is attributed to potential steps created by alternatively stacked Co and Ni oxide layers, which alter the electronic structure and facilitate electron transfer within the sample. This work, along with a previous demonstration of transition metal oxides with a specific distribution of charge for better OER activities, has shown the promise of this selective few-layer stacking methodology to improve the catalytic activity of a wide range of layered materials for the OER, and possibly for heterogeneous catalysis of other important redox reactions. These findings underscore the potential of layer-by-layer assembly as a design strategy for electrocatalysts in the OER and other redox reactions, encouraging future studies to explore atomic-scale organization for catalytic enhancement. These include work on other layered systems (such as layered double hydroxides) and in situ spectroscopic studies to monitor the oxidation state and oxygen character during catalysis.

## Experimental Section

### General

All chemicals were purchased from chemical vendors and used without further purification, except where otherwise noted. LiCoO_2_ was purchased from Thermo Scientific or synthesized (vide infra). LiNiO_2_ was purchased from Sigma-Aldrich or synthesized (vide infra). PXRD was performed on a Bruker D8 ADVANCE diffractometer using copper Kα radiation from a sealed tube or a Bruker Kappa PHOTON III DUO diffractometer with a Cu μS tube. The XRD data were processed using DIFFRAC.EVA software packages. ICP-OES samples were digested in 0.5 M hydroxylamine hydrochloride and analyzed to quantify elemental compositions using a Thermo Scientific iCAP 7000 Series ICP-OES. TEM samples were prepared by depositing one drop of NS suspension sample in water (100 mg/L) on a lacey carbon copper mesh TEM grid (400 mesh, Ted Pella) and allowed to air-dry. Images were collected using a JEOL JEM-1400 microscope operating at 120 kV. Electrochemical measurements were performed using a CH Instruments CHI660E electrochemical analyzer. X-ray photoelectron spectroscopy was performed on a Thermo Scientific K-Alpha+ XPS instrument at the University of Delaware.

### Synthesis of LiCoO_2_, LiNiO_2_, and LiCo_*x*_Ni_*y*_O_2_

The lithium transition metal oxides were prepared using a published protocol.^4,19^ In all cases, cotton pads were soaked in solutions of lithium nitrate (LiNO_3_) and transition metal nitrate (Co­(NO_3_)_2_, Ni­(NO_3_)_2_), purchased from Sigma-Aldrich. These were dissolved in deionized (DI) water with a 1:1 molar ratio and salt concentrations of 0.5 M. For example, a 50 mL solution containing 0.5 M LiNO_3_ and 0.5 M Co­(NO_3_)_2_ was used to synthesize LiCoO_2_. After soaking for 3 h, the cotton was taken out and squeezed to remove excessive liquid before being placed in a furnace and heated in air at a rate of 100 °C per hour to a temperature of 400 °C. After cooling, the resulting material was gently ground by a mortar and pestle and returned to the furnace to be heated at the same rate to a final temperature of 900 °C, which was maintained overnight. Subsequently, the product was taken from the furnace and cooled in air to room temperature. Finally, the product was washed with DI water, filtered, and air-dried. The identities of the solids were confirmed using PXRD and were compared to reported PXRD.^20^ From ICP-OES, the ratio of Li/Co in LiCoO_2_ is 1.006 to 1. The ratio of Li/Ni in LiNiO_2_ is 0.984 to 1.

### Synthesis of Single-Layer NS

The intercalated Li^+^ was removed by acid exchange by stirring 0.2 g of materials LiMO_2_ (M = Co, Ni) in a dilute HNO_3_ solution (0.1 M, 10 mL) for 2 days.^21^ The suspension was centrifuged at 7000 rpm/4000*g,* and the centrifugate was washed with water until the pH was between 6 and 7. Then, an aqueous solution of tetra-*n*-butylammonium hydroxide (TBAOH) was added to the solid to perform exfoliation. After stirring for 10 days, the TBAOH solution was removed by centrifugation at 14,000 rpm/16,000*g* for 15–30 min, and the precipitate was collected and washed by resuspending in ethanol and centrifuging again at 14,000 rpm/16,000*g* for 20 min, resuspending in water, and centrifuging again at 14,000/16,000*g* for an additional 30 min. The pellet was then dispersed again into water to form a colloidal solution of purified NS. The exfoliated NS supernatant was separated from incompletely dissolved colloidal particles by additional centrifugation at 7000 rpm/4000*g* for 15–25 min, and the NS supernatant was decanted. Note that NS will form a pellet upon centrifugation at 14,000 rpm/16,000*g* but will remain in the supernatant at 7000 rpm/4000*g*.

### Assembly of NS into Few-Layer Materials

This layer-by-layer assembly procedure was based upon the descriptions used in previous studies.^15,21^ First, a 1 cm × 1 cm sample of FTO was washed with acetone, ethanol, and water and dried in air. The substrate, dry FTO, was then immersed in a PEI solution (2.5 g/L) for 1 min to be coated. After rinsing with water three times and drying in air, the colloidal solution of NS (cobalt oxide, and nickel oxide) was drop-cast onto the PEI-precoated FTO to form an ultrathin NS layer. A paper towel was used to wick away the excessive solution before the film was washed with water and dried in air for 10 min. After that, a KCl solution (5 mM) was drop-cast onto the film, and then the excess KCl solution was wicked away with a paper towel, rinsed with water, and dried. Afterward, the procedure was repeated with an alternative coating of NS followed by K^+^. After up to six sheets were deposited, the materials were terminated by a K^+^ layer for charge-balance and a final water rinse. The fidelity of the deposition process was consistent with XPS analysis on substrates following deposition of Co-only, Ni-only, or mixed Co–Ni few-layer materials (Figure S7).

### Electrochemical Measurements

Electrochemical characterization of the various layered materials was performed using LSV with a sweep rate of 0.01 V/s in 1 M KOH using a Pt wire counter electrode, a saturated calomel electrode (SCE), or an Ag/AgCl electrode. The calibration of the reference electrode was checked against the redox couple of potassium ferricyanide in water. The working electrode was FTO (1 cm × 1 cm) (coated with the layered material of interest). We compared the effect of unpurified KOH electrolyte to that which had been purified to remove Fe using a published procedure.^28^ We noted a negligible difference in the LSV and thus conclude that contaminant iron does not influence the catalysis. Unpurified commercial KOH was used for the remainder of the experiments. The internal resistivity of the apparatus, as measured by impedance spectroscopy, was very low (0.5 ohms) due to the high-concentration electrolyte, and as such, the ∼5 mV ohmic drop correction was not applied. The measured potentials were converted to the RHE scale via the Nernst equation:

$$E_{\mathrm{RHE}}=E_{\mathrm{SCE}}+0.059\mathrm{pH}+{E^{{}^{\circ}}}_{\mathrm{ref}}$$

where *E*_RHE_ is the converted potential vs RHE, *E*_ref_ is the experimental potential measured against the reference electrode, and *E*°_ref_ = 0.2412 V for SCE at 25 °C vs RHE. The electrocatalysis was measured using LSV with a scan rate of 10 mV/s. The activity was normalized by dividing the current by the electrode area (∼1 cm^2^) to give currents in units of mA·cm^–2^.

In comparing individual samples for the OER, it is important to note that the activity of each catalyst is highly dependent upon the synthetic batch, and we observed, depending on batch, excellent or poor catalysts for both KNiO_2_ and KCoO_2_, due to differences in M^II^ concentration and defect density (Figures S4 and S6). To control for variability, the self-same synthetic batches were always compared to one another in mixed-metal and homogeneous catalysts for effective control experiments.

## Computational Methods

Computationally, the structures were initialized from bulk structures for LiCoO_2_^49^ and LiNiO_2_.^20^ To obtain the required potassium-intercalated structures, Li ions were replaced with K ions. Bulk KCoO_2_, KNiO_2_, same-layer KCo_0.5_Ni_0.5_O_2,_ and different-layer KCo_0.5_Ni_0.5_O_2_ structures were generated and fully optimized in their atomic positions and cell parameters. Furthermore, after testing different spin configurations (low-, intermediate-, and high-spin) for each model, we found that all bulk compounds are most stable in their low-spin configurations (see Figure S8).

Using the optimized bulk structures as a template, the following 24 atomic-layer computational surface structures were generated: pure cobalt oxide (Co/K)_6_, pure nickel oxide (Ni/K)_6_, and two alternately stacked nickel/cobalt oxides—one with Ni and Co in the same layer (Co0_.5_Ni_0.5_/K)_6_ and another with Ni and Co in different layers (Co/K/Ni/K)_3_. These structures were fully optimized in their atomic positions and cell parameters, exploiting their symmetry when possible. The computational slab models are shown in Figure 10. Surfaces were initialized from the bulk structures by performing a slab cut perpendicular to the (001) plane to generate the few-layer models. We assigned a vacuum of 500 Å (CRYSTAL17’s default vacuum size for slab calculations) in the [001] direction for all surface models to avoid self-interactions arising from periodic boundary conditions. The models are equivalent to the experimental structures, starting with a transition metal oxide layer and ending in a potassium layer. We did not initiate the layered structures from the FTO glass used in the experimental procedure, as it is impossible to model nonperiodic glass structures at this level of theory. Several low-, intermediate-, and high-spin configurations for the slabs were tested. In this case, it was found that self-consistent field (SCF) convergence criteria were only met in a reasonable number of steps (≤800 SCF steps) for either low or intermediate spin order, depending on the atomic configuration.

**Figure 10:**
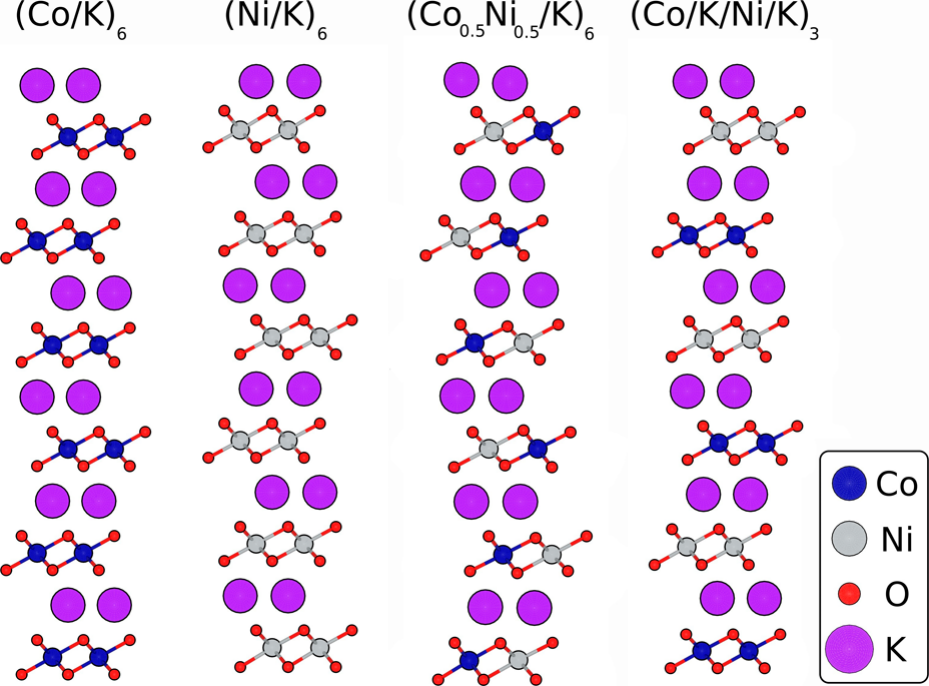
Slab models for (Co/K)_6_, (Ni/K)_6_, (Co_0.5_Ni_0.5_/K)_6_, and (Co/K/Ni/K)_3_.

Geometry optimizations and electronic structures for these materials were computed by using unrestricted DFT (UDFT). Calculations were performed using the Heyd–Scuseria–Ernzerhof (HSE06) exchange–correlation functional,^29^ as implemented in the CRYSTAL17 code.^30^ We chose HSE06 due to its high reliability in accurately predicting structural and electronic properties.^31^ Furthermore, CRYSTAL17 uses Gaussian-type orbitals, which allow efficient implementation of Post-Hartree–Fock methods. All unit cell optimizations and single-point calculations were performed using triple-ζ with polarization quality (TZVP) Gaussian basis sets for K, Co, Ni, and O atoms.^32^ The semi-empirical Grimme-D3 dispersion correction was used to estimate van der Waals forces.^33^ The direct inversion of the iterative subspace convergence accelerator was used for all optimizations and single-point energy calculations.^34^

The convergence threshold for energy, forces, and electron density was 10^–7^ au for all parameters. The reciprocal space for all of the structures was sampled using a Γ-centered Monkhorst–Pack scheme with a resolution of around 2π × 1/60 Å^–1^. During geometry optimization and single-point energy calculations, the SPIN and SPINLOCK keywords were used to specify the unrestricted wave functions and total spin of the transition metals. Furthermore, the ATOMSPIN keyword was used to specify unpaired electrons for the individual transition metals. High-symmetry *k*-point coordinates for the bulk materials’ band structures were obtained using the SeeK-path software^35^ and are unique to each geometry. DOS calculations were performed for all bands and are shown in the −10 to 10 eV range (around the Fermi level) for bulk calculations and −10 to 0 eV range (with respect to vacuum) for slab calculations. The absolute band alignment was performed by running single-point calculations using the optimized structures, with ghost atoms in the vicinity of the slabs’ outermost layers for a better description of the electrostatic potential in vacuum. Slab DOS were shifted relative to the vacuum level to obtain a more accurate description of the materials’ absolute band alignment. The vacuum level was defined as the asymptotic value of the plane-averaged electrostatic potential, sufficiently far from the slab (∼40 Å in our calculations).

## Supplementary Material



## Notes

Additional electrochemical data, electron micrograms, and additional computational results (PDF)

## ∥

Department of Materials Science and Engineering, North Carolina State University, 3002 Engineering Building 1, 911 Partners Way, Raleigh, North Carolina 27695, United States

## Abbreviations

OER: oxygen evolution reaction
DOS: density of states
VBM: valence band maximum
CBM: conduction band minimum
TEM: transmission electron microscopy
PXRD: powder X-ray diffraction
ICP-OES: inductively coupled plasma-optical emission spectroscopy
PEI: polyethylenimine
FTO: fluorine-doped tin oxide
TBAOH: tetrabutylammonium hydroxide
NS: nanosheet
RHE: reversible hydrogen electrode
LSV: linear sweep voltammetry
DFT: density functional theory
UDFT: unrestricted density functional theory
SOE: standard oxygen electrode
SOE + OP: standard oxygen electrode + overpotential
SCE: saturated calomel electrode

## References

[1] Nocera D. G. (2012). The Artificial
Leaf. Acc. Chem. Res..

[2] Liu P. F., Yang S., Zhang B., Yang H. G. (2016). Defect-Rich
Ultrathin Cobalt–Iron Layered Double Hydroxide for Electrochemical
Overall Water Splitting. ACS Appl. Mater. Interfaces.

[3] Zhu J., Chen J., Li X., Luo K., Xiong Z., Zhou Z., Zhu W., Luo Z., Huang J., Li Y. (2024). Steering surface reconstruction of
hybrid metal oxides for efficient oxygen evolution reaction in water
splitting and zinc-air batteries. Journal of
Energy Chemistry.

[4] Lu Z., Wang H., Kong D., Yan K., Hsu P.-C., Zheng G., Yao H., Liang Z., Sun X., Cui Y. (2014). Electrochemical tuning of layered lithium transition
metal oxides
for improvement of oxygen evolution reaction. Nat. Commun..

[5] Wang P. P., Fu P., Ma J. P., Gao Y. Y., Li Z., Wang H., Fan F. T., Shi J. Y., Li C. (2021). Ultrathin
Cobalt Oxide Interlayer Facilitated Hole Storage for Sustained Water
Oxidation over Composited Tantalum Nitride Photoanodes. ACS Catal..

[6] Thenuwara A. C., Shumlas S. L., Attanayake N. H., Cerkez E. B., McKendry I. G., Frazer L., Borguet E., Kang Q., Zdilla M. J., Sun J. (2015). Copper-Intercalated
Birnessite as a Water Oxidation Catalyst. Langmuir.

[7] Thenuwara A. C., Cerkez E. B., Shumlas S. L., Attanayake N. H., McKendry I. G., Frazer L., Borguet E., Kang Q., Remsing R. C., Klein M. L. (2016). Nickel
Confined in the
Interlayer Region of Birnessite: an Active Electrocatalyst for Water
Oxidation. Angew. Chem., Int. Ed..

[8] McKendry I. G., Kondaveeti S. K., Shumlas S. L., Strongin D. R., Zdilla M. J. (2015). Decoration
of the layered manganese oxide birnessite with Mn­(ii/iii) gives a
new water oxidation catalyst with fifty-fold turnover number enhancement. Dalton Transactions.

[9] Najafpour M. M., Ehrenberg T., Wiechen M., Kurz P. (2010). Calcium Manganese­(III)
Oxides (CaMn2O4·x H2O) as Biomimetic Oxygen-Evolving Catalysts. Angew. Chem., Int. Ed..

[10] McKendry I. G., Thenuwara A. C., Shumlas S. L., Peng H., Aulin Y. V., Chinnam P. R., Borguet E., Strongin D. R., Zdilla M. J. (2018). Systematic
Doping of Cobalt into Layered Manganese Oxide Sheets Substantially
Enhances Water Oxidation Catalysis. Inorg. Chem..

[11] McKendry I. G., Mohamad L. J., Thenuwara A. C., Marshall T., Borguet E., Strongin D. R., Zdilla M. J. (2018). Synergistic In-Layer Cobalt Doping
and Interlayer Iron Intercalation into Layered MnO2 Produces an Efficient
Water Oxidation Electrocatalyst. ACS Energy
Letters.

[12] Ding R., Yasini P., Peng H., Perdew J. P., Borguet E., Zdilla M. J. (2021). Reimagining the eg1 Electronic State
in Oxygen Evolution
Catalysis: Oxidation-State-Modulated Superlattices as a New Type of
Heterostructure for Maximizing Catalysis. Adv.
Energy Mater..

[13] Peng H., McKendry I. G., Ding R., Thenuwara A. C., Kang Q., Shumlas S. L., Strongin D. R., Zdilla M. J., Perdew J. P. (2017). Redox properties of birnessite from a defect perspective. Proc. Natl. Acad. Sci. U. S. A..

[14] Bhullar R.
K., Zdilla M. J., Klein M. L., Remsing R. C. (2021). Effect of water frustration on water
oxidation catalysis
in the nanoconfined interlayers of layered manganese oxides birnessite
and buserite. Journal of Materials Chemistry
A.

[15] Kang Q., Vernisse L., Remsing R. C., Thenuwara A. C., Shumlas S. L., McKendry I. G., Klein M. L., Borguet E., Zdilla M. J., Strongin D. R. (2017). Effect of Interlayer
Spacing on the
Activity of Layered Manganese Oxide Bilayer Catalysts for the Oxygen
Evolution Reaction. J. Am. Chem. Soc..

[16] Elmaci G., Ozgenc G., Kurz P., Zumreoglu-Karan B. (2020). Enhanced water
oxidation performances of birnessite and magnetic birnessite nanocomposites
by transition metal ion doping. Sustainable
Energy & Fuels.

[17] Jiang H., Dong H., Liu Y., Wan Q., Pan F., Zhang S., Yang Z., Chen Y., Chen L., Zheng X. (2025). Reconstructed Hydroxyl
Coordination Field Enhances Mass Transfer for Efficient Electrocatalytic
Water Oxidation. Small.

[18] Maitra U., Naidu B. S., Govindaraj A., Rao C. N. R. (2013). Importance of trivalency and the eg1 configuration
in the photocatalytic oxidation of water by Mn and Co oxides. Proc. Natl. Acad. Sci. U. S. A..

[19] Deshazer H. D., Mantia F. L., Wessells C., Huggins R. A., Cui Y. (2011). Synthesis
of Nanoscale Lithium-Ion Battery Cathode Materials Using a Porous
Polymer Precursor Method. J. Electrochem. Soc..

[20] Laubach S., Laubach S., Schmidt P. C., Ensling D., Schmid S., Jaegermann W., Thißen A., Nikolowski K., Ehrenberg H. (2009). Changes in
the crystal and electronic structure of
LiCoO2 and LiNiO2 upon Li intercalation and de-intercalation. Phys. Chem. Chem. Phys..

[21] Wang, Omomo Y., Sakai N., Fukuda K., Nakai I., Ebina Y., Takada K., Watanabe M., Sasaki T. (2003). Fabrication and Characterization
of Multilayer Ultrathin Films of Exfoliated MnO2 Nanosheets and Polycations. Chem. Mater..

[22] Orman H. J., Wiseman P. J. (1984). Cobalt­(III) lithium
oxide, CoLiO2: structure refinement by powder neutron diffraction. Acta Crystallographica Section C.

[23] Guidelli R., Compton R. G., Feliu J. M., Gileadi E., Lipkowski J., Schmickler W., Trasatti S. (2014). Defining the transfer coefficient in electrochemistry:
An assessment (IUPAC Technical Report). Pure
Appl. Chem..

[24] Huang H., Chang Y.-C., Huang Y.-C., Li L., Komarek A. C., Tjeng L. H., Orikasa Y., Pao C.-W., Chan T.-S., Chen J.-M. (2023). Unusual double ligand holes as catalytic active sites
in LiNiO2. Nat. Commun..

[25] Gardner G., Al-Sharab J., Danilovic N., Go Y. B., Ayers K., Greenblatt M., Charles Dismukes G. (2016). Structural basis for differing electrocatalytic
water oxidation by the cubic, layered and spinel forms of lithium
cobalt oxides. Energy Environ. Sci..

[26] Narayanan, H. ; Viswanathan, B. ; Krishnamurthy, K. R. ; Nair, H. Chapter 12 - Hydrogen from photo-electrocatalytic water splitting. In Solar Hydrogen Production; Calise, F. ; D’Accadia, M. D. ; Santarelli, M. ; Lanzini, A. ; Ferrero, D. , Eds.; Academic Press, 2019; pp 419–486.

[27] Minaev B. F. (2001). Spin effects
in activation of hydrocarbons: The role of triplet states in catalysis. J. Mol. Catal. A: Chem..

[28] Trotochaud L., Young S. L., Ranney J. K., Boettcher S. W. (2014). Nickel–Iron
Oxyhydroxide Oxygen-Evolution Electrocatalysts: The Role of Intentional
and Incidental Iron Incorporation. J. Am. Chem.
Soc..

[29] Heyd J., Scuseria G. E., Ernzerhof M. (2003). Hybrid functionals
based on a screened Coulomb potential. J. Chem.
Phys..

[30] Dovesi R., Erba A., Orlando R., Zicovich-Wilson C. M., Civalleri B., Maschio L., Rérat M., Casassa S., Baima J., Salustro S. (2018). Quantum-mechanical
condensed matter simulations with CRYSTAL. WIREs
Computational Molecular Science.

[31] Heyd J., Scuseria G. E. (2004). Efficient hybrid density functional
calculations in
solids: Assessment of the Heyd–Scuseria–Ernzerhof screened
Coulomb hybrid functional. J. Chem. Phys..

[32] Vilela
Oliveira D., Laun J., Peintinger M. F., Bredow T. (2019). BSSE-correction scheme for consistent gaussian basis
sets of double- and triple-zeta valence with polarization quality
for solid-state calculations. J. Comput. Chem..

[33] Grimme S., Antony J., Ehrlich S., Krieg H. (2010). A consistent and accurate
ab initio parametrization of density functional dispersion correction
(DFT-D) for the 94 elements H-Pu. J. Chem. Phys..

[34] Pulay P. (1980). Convergence acceleration of iterative
sequences. the case of scf iteration. Chem.
Phys. Lett..

[35] b Togo, A. ; Tanaka, I. A Software Library for Crystal Symmetry Search. 2018, ArXiv.

