# Supplementary material for: Enhanced Activity in Layered Metal-Oxide-Based Oxygen Evolution Catalysts by Layer-by-Layer Modulation of Metal-Ion Identity
Source: ACS Catal. 2025 Nov 24;15(23):20204–15. doi: 10.1021/acscatal.5c02788 (PMC12687313; doi:10.1021/acscatal.5c02788)
Supplement: Supplementary file 1 [file cs5c02788_si_001.pdf]

Supporting Information for:

Enhanced activity in layered-metal-oxide-based oxygen evolution catalysts by layer-by-layer modulation of metal ion identity.

Ran Ding,[†§1] Daniel Maldonado-Lopez,[§2] Jacob E. Henebry,[1] Jose Mendoza-Cortes,[2,3,*] Michael J. Zdilla[1,*]

[1]Deparment of Chemistry, Temple University, 1901 N. 13th St., Philadelphia, PA

[2]Department of Chemical Engineering & Materials Science, Michigan State University, East Lansing, MI 48824, USA

[3]Department of Physics & Astronomy, Michigan State University, East Lansing, Michigan 48824, USA

[§]These authors contributed equally.

Corresponding Author: * mzdilla@temple.edu, jmendoza@msu.edu

[†]Present Addresses. Department of Materials Science and Engineering, North Carolina State University, 3002 Engineering Building 1, 911 Partners Way, Raleigh NC 27695

Contents

Contents 2

Transmission Electron Microscopy 3

Figure S1. TEM images of LiCoO2 3

Figure S2. TEM images of $LiNiO_2$ 3

Figure S3. TEM images of LiCoxNi1-xO2 4

Additional Electrochemical Data 5

Figure S4. Linear sweep voltammetry of different sample batches 5

Figure S5. Chronoamperometry 6

Table S1. Summary of mixed cobalt and nickel layered catalysts 7

X-ray Photoelectron Spectroscopy 8

Additional Computational Results 10

Transmission Electron Microscopy

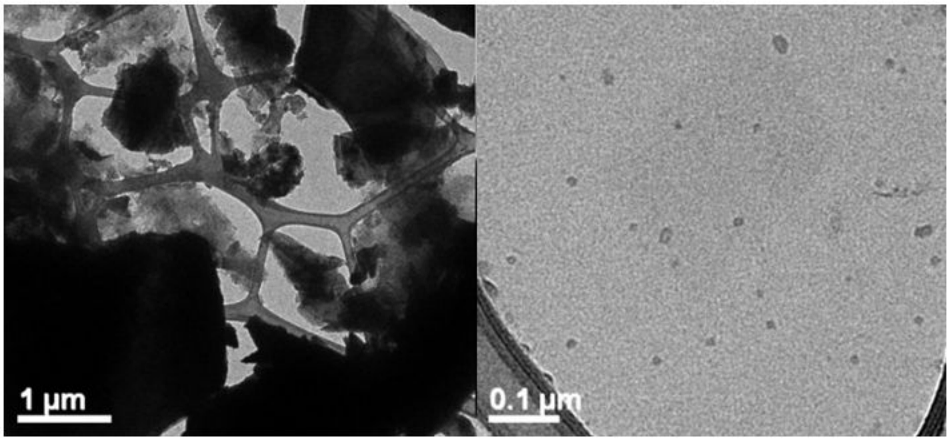

Figure S1. TEM images of $LiCoO_2$: Bulk (left) and single layer nanosheets (right).

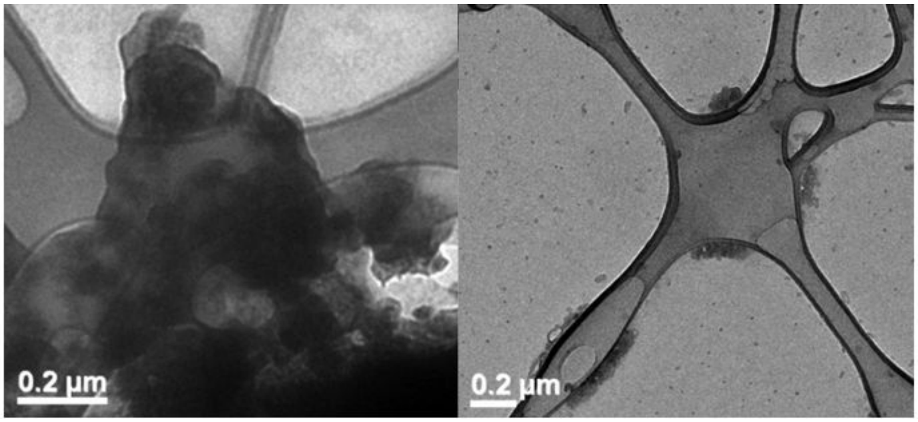

Figure S2. TEM images of $LiNiO_2$: Bulk (left) and single layer nanosheets (right).

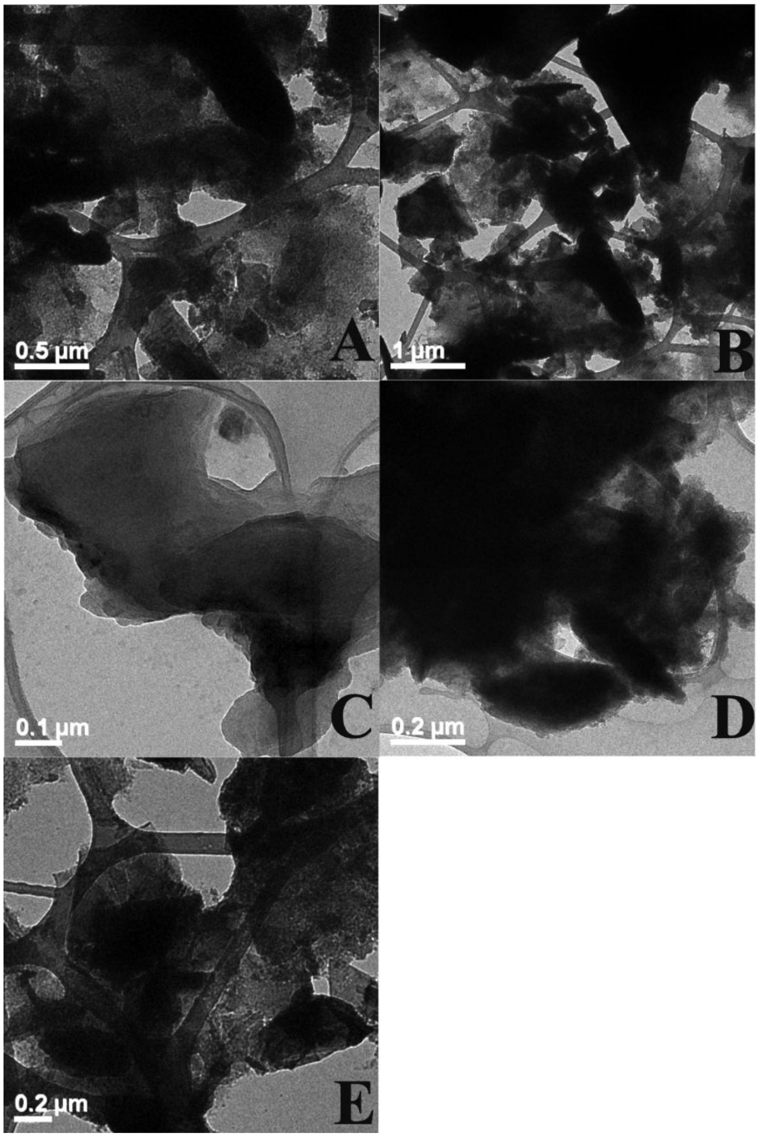

Figure S3. TEM images of $LiCo_xNi_{(1-x)}O_2$. $x = 1$ (A), 0.66 (B), 0.50 (C), 0.33 (D) and 0 (E).

Additional Electrochemical Data

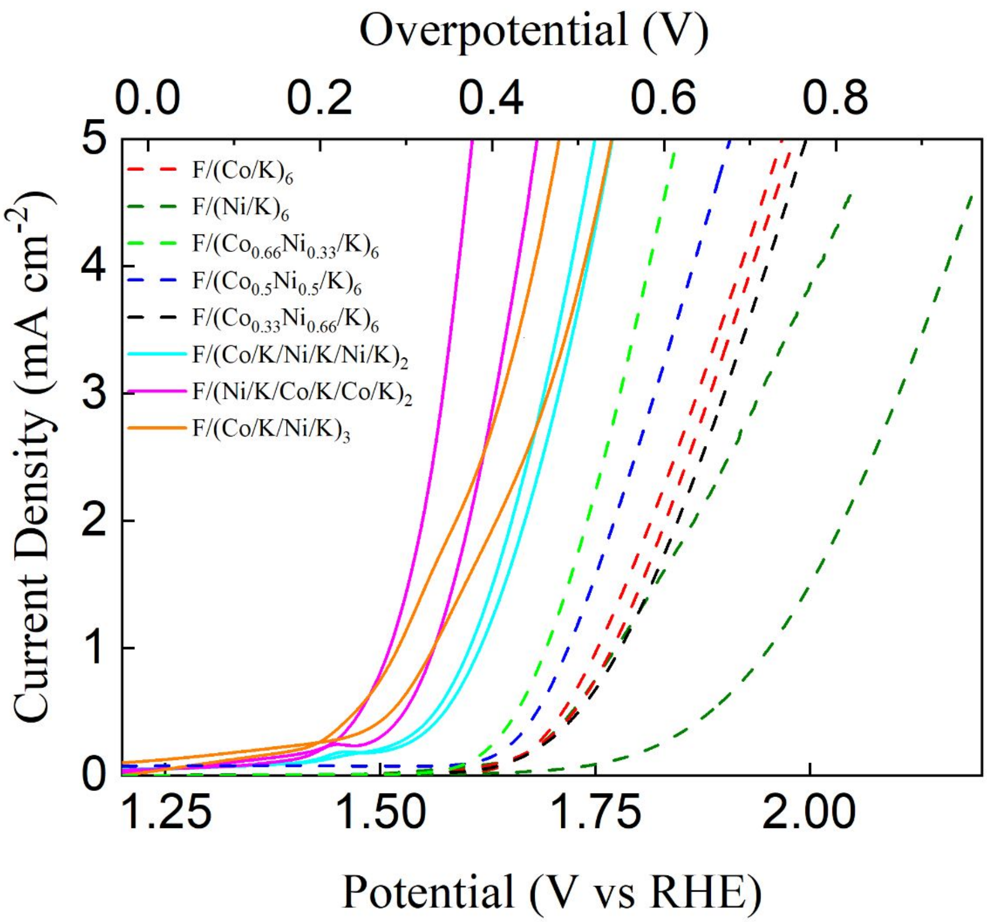

Figure S4. Linear sweep voltammetry of different sample batches of few-layer catalysts, illustrating slight variability (due presumably to slight differences in coverage or layer adhesion), but a robust overall trend. Each curve represents an average of three electrodes prepared from the same precursor batch. Multiple traces of the same color represent the highest- and lowest-overpotential samples we observed, so as to show the full range of catalytic activity across the samples due to differences in defect density and catalytic activity.

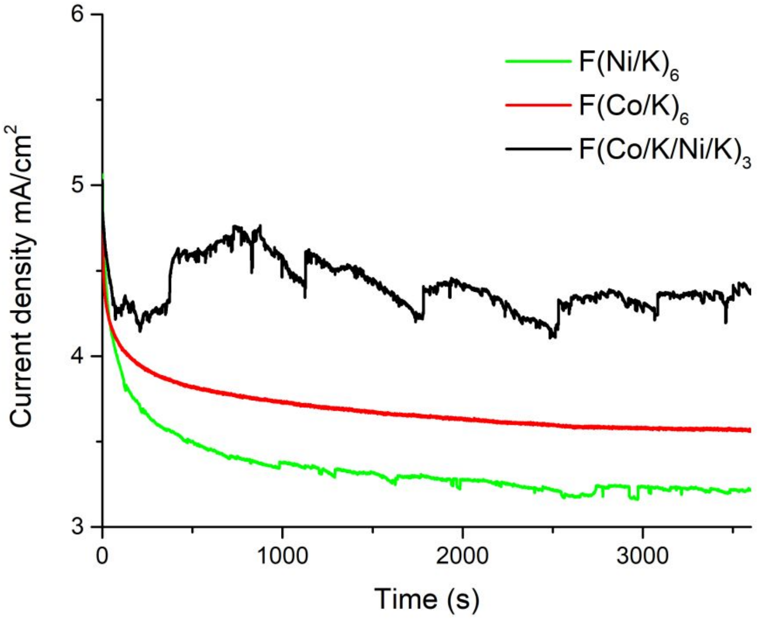

Figure S5. Chronoamperometry of the oxygen evolution reaction with F(Co/K)$_6$ at a potential of 1.7 V versus RHE (red); F(Ni/K)$_6$ at a potential of 1.9 V versus RHE (green); F(Co/K/Ni/K)$_3$ at a potential of 1.6 V versus RHE (black). Noise in the plots is the result of bubble formation on the electrodes.

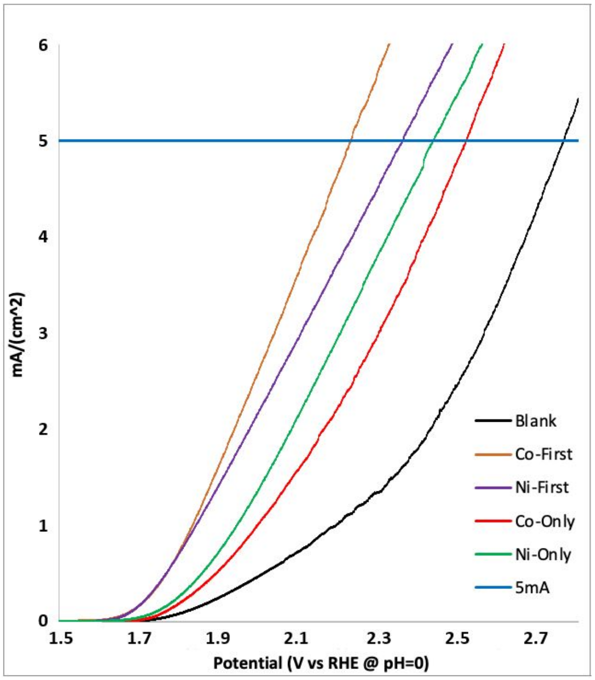

Figure S6. Linear sweep voltammetry of F/(Ni/K)$_6$ (—), F/(Co/K)$_6$(—), F/(Ni/K/Co/K)$_3$(—), and F/(Co/K/Ni/K)$_3$(—). $KNiO_2$ catalyst was slightly superior to the $KCoO_2$ catalyst, but the alternating system with Co first was superior to all other arrangements.

Table S1. Summary of mixed cobalt and nickel layered catalysts by electrochemistry.

Sample	Overpotential (mV)	Tafel Slope (mV/dec)
$F/(Co/K)_6$	705	390
$F/(Ni/K)_6$	800	400
$F/(Co_{0.66}Ni_{0.33}/K)_6$	600	173
$F/(Co_{0.5}Ni_{0.5}/K)_6$	650	314
$F/(Co_{0.33}Ni_{0.66}/K)_6$	740	245
$F/(Co/K/Ni/K)_3$	460	280
$F/(Co/K/Ni/K/Ni/K)_2$	500	175
$F/(Ni/K/Co/K/Co/K)_2$	370	130

X-ray Photoelectron Spectroscopy

To confirm controlled layer deposition using the described dipping method, we employed X-ray photoelectron spectroscopy. We observed signal for whichever layered materials were used in the dip, though signals are weak due to the atomically thin layer and the deeper escape depth of the photoelectrons in comparison to the layer thickness. We observed that when samples are alternatively dipped in CoO_2 nanosheets with NiO_2 sheets, both signals appear. Finally, postmortem XPS shows the retention of most or all of the CoO_2 and $NiO_{2,}$ though some loss of intensity due to leeching is noted.

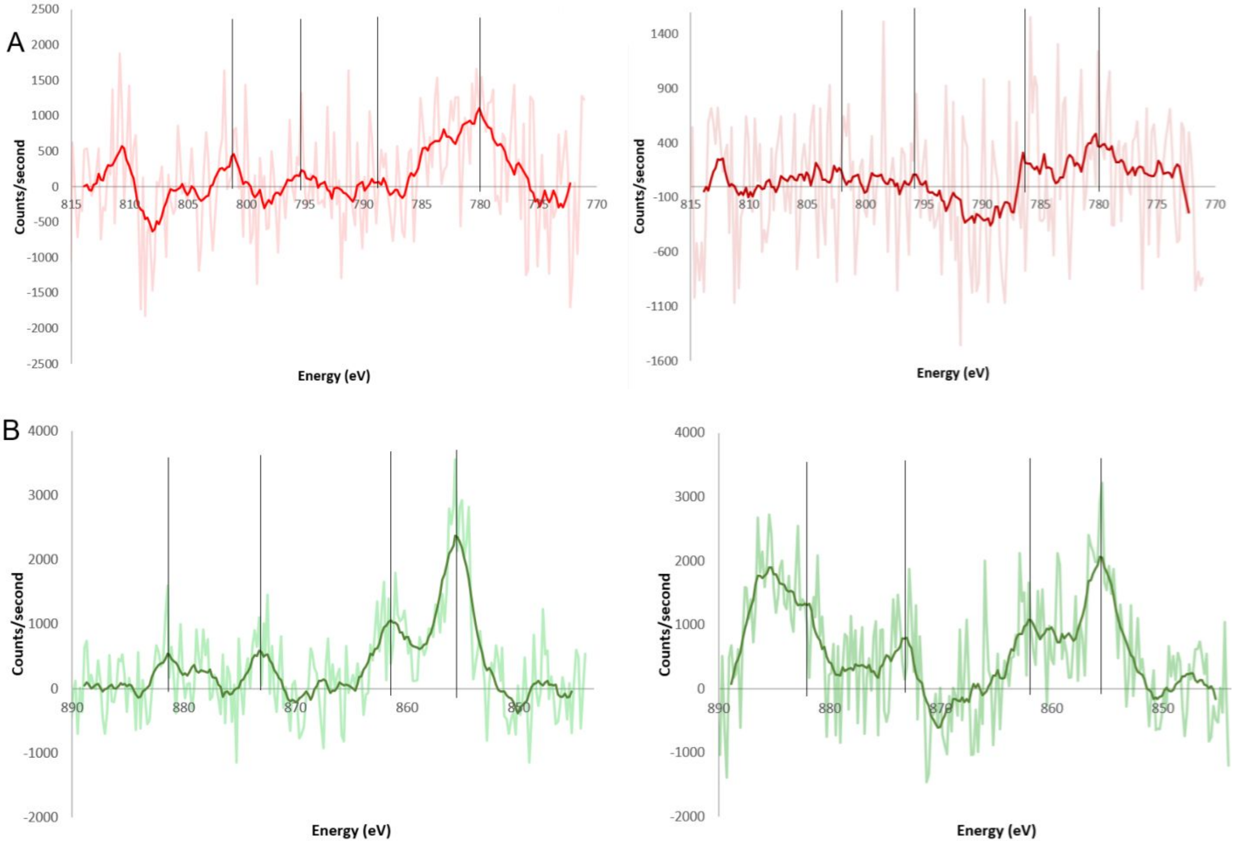

Figure S7. 2p XPS spectra of few-layered KMO_2 catalysts deposited on PEI-coated FTO. Co 2p $^{3}/_{2}$ peaks at 780eV w/ satellite 785 – 790, Co 2p ½ peaks at 796eV w/ satellite at 802 – 805. Ni 2p $^{3}/_{2}$ peaks at 855 eV w/ satellite at 865 eV, Ni 2p½ peaks at 874 eV w/ sattelite at 883 eV. (A) Co XPS of $F(Co/K)_6$ before and after catalysis. (B) Ni XPS of $F(Ni/K)_6$ before and after catalysis.

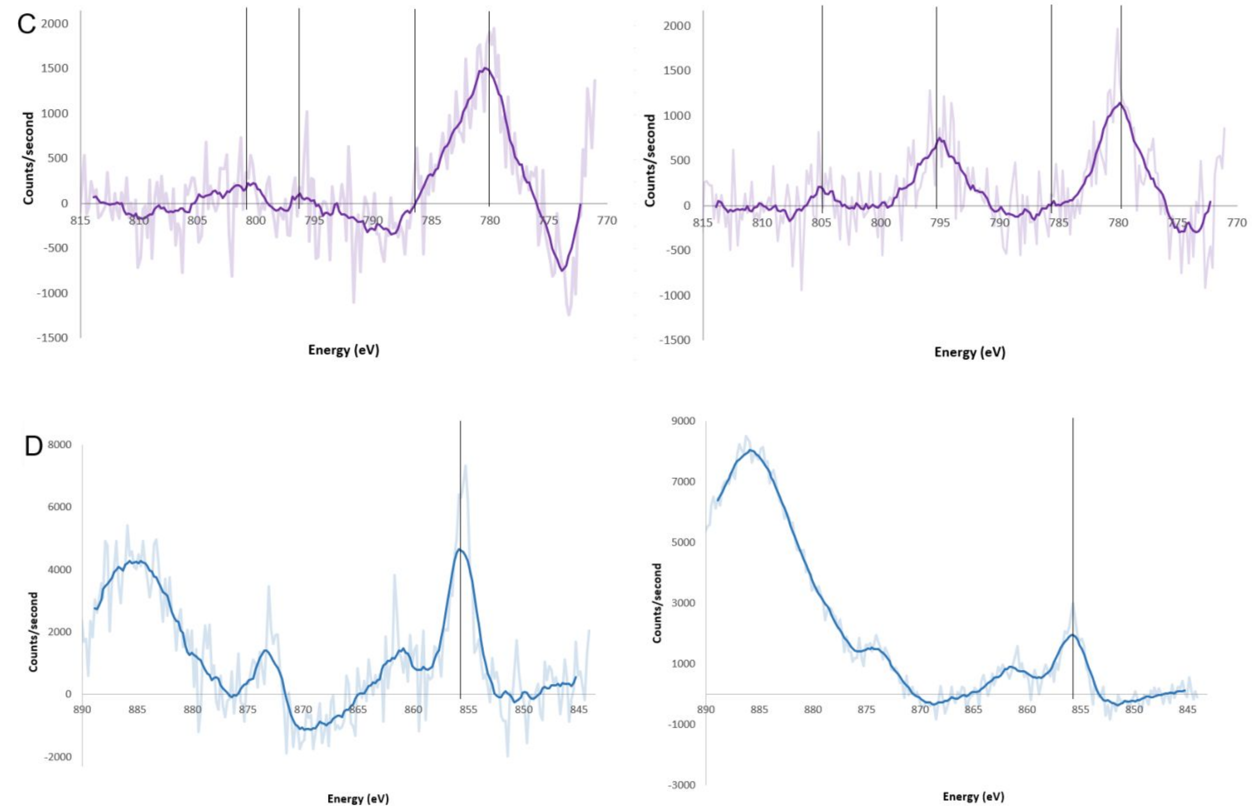

Figure S7 (continued). 2p XPS spectra of few-layered KMO_2 catalysts deposited on PEI-coated FTO. Co 2p $^{3}/_{2}$ peaks at 780eV w/ satellite 785 – 790, Co 2p ½ peaks at 796eV w/ satellite at 802 – 805. Ni 2p $^{3}/_{2}$ peaks at 855 eV w/ satellite at 865 eV, Ni 2p½ peaks at 874 eV w/ sattelite at 883 eV. (C) Co XPS of F(Co/K/Ni/K)$_3$ before and after catalysis. (D) Ni XPS of F(Co/K/Ni/K)$_3$ before and after catalysis.

Additional Computational Results

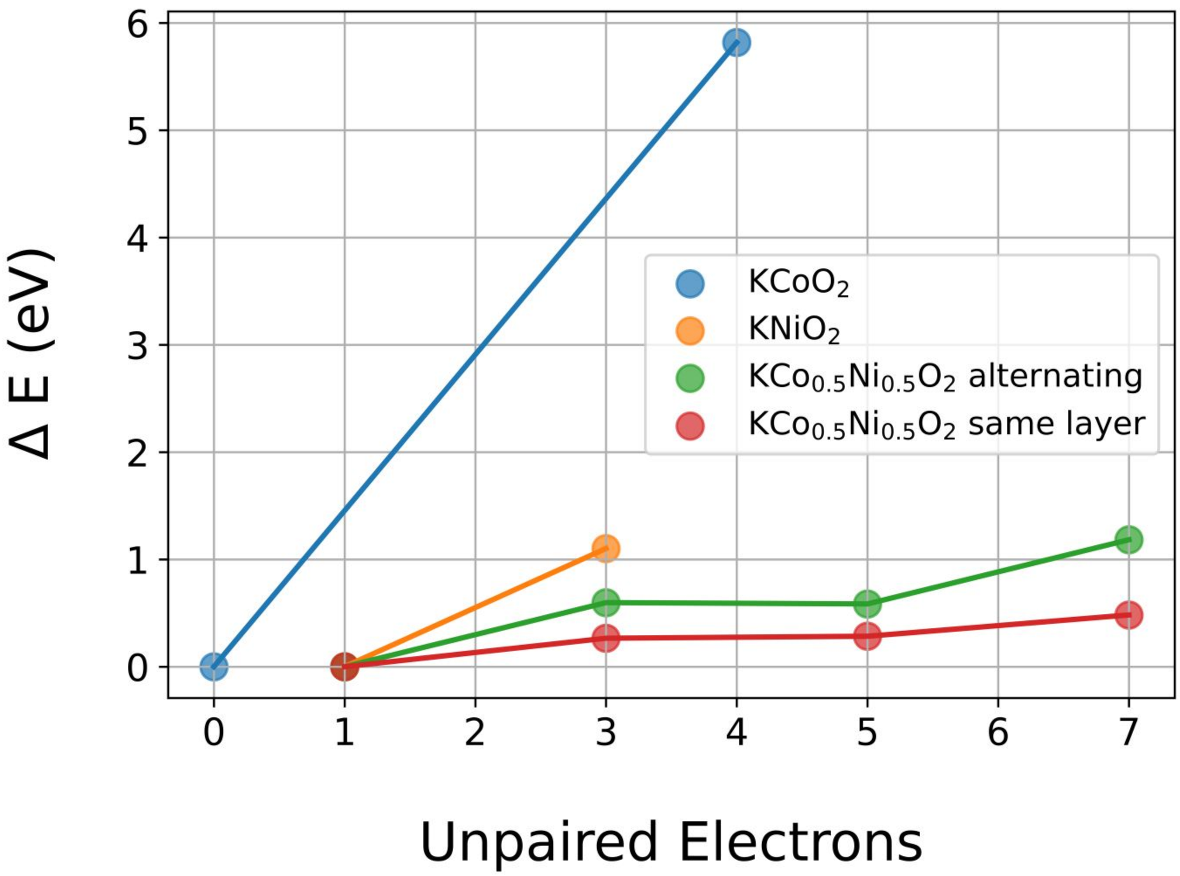

Figure S8. Energy comparison for different spin states. Energy difference between converged bulk structures $KCoO_2$, $KNiO_2$, alternating $KCo_{0.5}Ni_{0.5}O_2$, and same-layer $KCo_{0.5}Ni_{0.5}O_2$ at low-spin, intermediate-spin, and high-spin configurations. $KCoO_2$ and $KNiO_2$ are defined with only one transition metal in their unit cell. Therefore, they exclusively present high- and low-spin configurations.

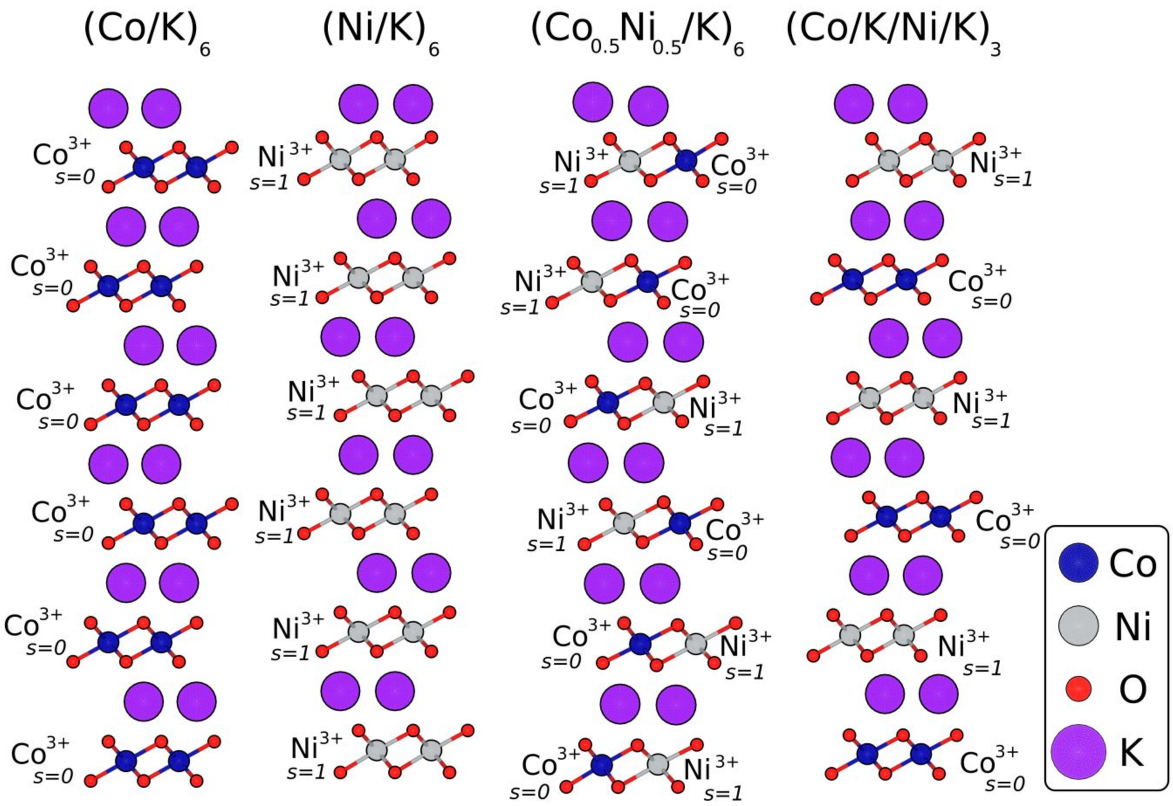

Figure S9. Oxidation states and unpaired electrons for Co and Ni atoms in the $(Co/K)_6$, $(Ni/K)_6$, $(Co_{0.5}Ni_{0.5}/K)_6$, and $(Co/K/Ni/K)_3$ few-layer slab structures. This information was taken from the α-β Mulliken Population Analysis of the converged models. Since oxygen and potassium ions present negligible unpaired spins, they are assumed to have a charge of 2- and 1+, respectively.

For Figure S7, it is important to point out that Mulliken Population Analysis gives a rough estimation of unpaired electrons. In general, this analysis is accurate for elements containing only *s*- and *p*-orbitals. For the $(Ni/K)_6$, $(Co_{0.5}Ni_{0.5}/K)_6$, and $(Co/K/Ni/K)_3$ slabs, the α−β spin distribution was slighly different (by ~0.2−0.5 unpaired e^-) for outer Ni atoms, compared to inner Ni atoms. Therefore, Ni^{2+} and Ni^{4+} ions could be present in the structures. Magnetic moment calculations are necessary to confirm the spin structure and oxidation states.

Crytallographic Structures in CIF format

*Note that **slab** CIF files have a c lattice parameter of 40 or 60 Å for better visualization in the VESTA software. However, calculations were carried out with a cell length of 500 Å in the c-(Co/K)$_6$ CIF file direction to avoid interactions between periodic images of the slab. This spacing is automatically assigned by the CRYSTAL17 software when setting up slab calculations.*

(Co/K)$_6$ CIF file

```
_cell_length_a                          2.94109216
_cell_length_b                          2.94122886
_cell_length_c                          40.00000000
_cell_angle_alpha                       90.000000
_cell_angle_beta                        90.000000
_cell_angle_gamma                       120.001700
_symmetry_space_group_name_H-M          'P 1'
_symmetry_Int_Tables_number             1

loop_
_symmetry_equiv_pos_as_xyz
   'x, y, z'

loop_
   _atom_site_label
   _atom_site_type_symbol
   _atom_site_fract_x
   _atom_site_fract_y
   _atom_site_fract_z
 K001   K  0.0010945638  0.0001274167  0.4296193442
 O002   O  0.3336179058  -0.3341164605  -0.3867563365
 O003   O  -0.3324388119  0.3333235225  0.3821493909
CO004  CO  0.3343454380  -0.3333010517  0.3579180276
CO005  CO  -0.3331403391  0.3325370476  -0.3661572098
 O006   O  0.0009453688  -0.0000148348  0.3342076611
 O007   O  0.0004371792  -0.0006495237  -0.3413562799
 K008   K  0.3333898515  -0.3341735793  -0.2932054120
 K009   K  -0.3324988094  0.3331872981  0.2851768318
 O010   O  0.3340008444  -0.3335678848  0.2372027708
 O011   O  -0.3334609474  0.3323341291  -0.2441915144
CO012  CO  0.0006960685  -0.0002191606  0.2132572233
CO013  CO  -0.0001251722  -0.0009867941  -0.2204805217
 O014   O  0.3332128236  -0.3343233576  -0.1965339844
 O015   O  -0.3326653461  0.3330999537  0.1895612784
```

```
 K016   K  0.3338981337  -0.3336965223  0.1405911138
 K017   K  -0.3332945158  0.3324807790  -0.1485594954
 O018   O  0.0003993688  -0.0004810665  0.0926244132
 O019   O  0.0001624304  -0.0007062869  -0.0995906472
CO020  CO  0.3335269645  -0.3340227553  -0.0758933928
CO021  CO  -0.3329287863  0.3328545087  0.0686809149
 O022   O  0.3337298725  -0.3338126751  0.0449836252
 O023   O  -0.3331663703  0.3326272935  -0.0519483649
 K024   K  0.0003182850  -0.0005949956  -0.0039828113
```

$(Ni/K)_6$ CIF file – Note: Optimized in P3m1 symmetry (space group 156)

```
_cell_length_a                         3.03533944
_cell_length_b                         3.03533944
_cell_length_c                         40.00000000
_cell_angle_alpha                      90.000000
_cell_angle_beta                       90.000000
_cell_angle_gamma                      120.000000
_symmetry_space_group_name_H-M         'P 1'
_symmetry_Int_Tables_number            1

loop_
_symmetry_equiv_pos_as_xyz
   'x, y, z'

loop_
   _atom_site_label
   _atom_site_type_symbol
   _atom_site_fract_x
   _atom_site_fract_y
   _atom_site_fract_z
 K001   K  -0.3333333333  0.3333333333  0.4057960009
 O002   O  0.3333333333  -0.3333333333  0.3645391241
NI003  NI  0.0000000000  0.0000000000  0.3376022201
 O004   O  -0.3333333333  0.3333333333  0.3131023641
 K005   K  0.3333333333  -0.3333333333  0.2649300997
 O006   O  0.0000000000  0.0000000000  0.2163510160
NI007  NI  -0.3333333333  0.3333333333  0.1922869317
 O008   O  0.3333333333  -0.3333333333  0.1681468771
 K009   K  0.0000000000  0.0000000000  0.1198644760
 O010   O  -0.3333333333  0.3333333333  0.0715868951
NI011  NI  0.3333333333  -0.3333333333  0.0474663059
 O012   O  0.0000000000  0.0000000000  0.0232821193
 K013   K  -0.3333333333  0.3333333333  -0.0249733103
 O014   O  0.3333333333  -0.3333333333  -0.0732801856
NI015  NI  0.0000000000  0.0000000000  -0.0974138301
```

```
 O016   O  -0.3333333333  0.3333333333  -0.1215694095
 K017   K  0.3333333333  -0.3333333333  -0.1698489267
 O018   O  0.0000000000  0.0000000000  -0.2181497930
NI019  NI  -0.3333333333  0.3333333333  -0.2423100615
 O020   O  0.3333333333  -0.3333333333  -0.2666156960
 K021   K  0.0000000000  0.0000000000  -0.3144340110
 O022   O  -0.3333333333  0.3333333333  -0.3632507796
NI023  NI  0.3333333333  -0.3333333333  -0.3871699231
 O024   O  0.0000000000  0.0000000000  -0.4086018789
```

(Co/K/Ni/K)$_3$ CIF file

```
_cell_length_a                         2.95807100
_cell_length_b                         2.95888900
_cell_length_c                         40.00000000
_cell_angle_alpha                      90.000000
_cell_angle_beta                       90.000000
_cell_angle_gamma                      118.821700
_symmetry_space_group_name_H-M         'P 1'
_symmetry_Int_Tables_number            1

loop_
_symmetry_equiv_pos_as_xyz
   'x, y, z'

loop_
   _atom_site_label
   _atom_site_type_symbol
   _atom_site_fract_x
   _atom_site_fract_y
   _atom_site_fract_z
 O001   O  0.0714980000  -0.0736930000  0.4059572500
CO002  CO  0.4056390000  -0.4080820000  0.3856705250
 O003   O  -0.2596400000  0.2574510000  0.3612481250
 K004   K  0.0801300000  -0.0815350000  0.3127136750
 O005   O  0.4106040000  -0.4123300000  0.2644902500
NI006  NI  -0.2750030000  0.2740120000  0.2397431000
 O007   O  0.0394120000  -0.0398030000  0.2150116500
 K008   K  0.3716410000  -0.3719370000  0.1668135750
 O009   O  -0.2911460000  0.2906390000  0.1185241750
CO010  CO  0.0433290000  -0.0438760000  0.0949799250
 O011   O  0.3777580000  -0.3784700000  0.0714357500
 K012   K  -0.2845180000  0.2835990000  0.0231551750
 O013   O  0.0472640000  -0.0481640000  -0.0250359500
NI014  NI  0.3619630000  -0.3616610000  -0.0497636500
```

```
 O015   O  -0.3233890000  0.3247890000  -0.0744933250
 K016   K  0.0088510000  -0.0073210000  -0.1226762500
 O017   O  0.3461230000  -0.3448230000  -0.1709703250
CO018  CO  -0.3194000000  0.3206140000  -0.1945113750
 O019   O  0.0150630000  -0.0139270000  -0.2180575750
 K020   K  0.3527270000  -0.3517130000  -0.2663490500
 O021   O  -0.3140210000  0.3150210000  -0.3147987000
NI022  NI  0.0053940000  -0.0039410000  -0.3394442750
 O023   O  0.3235410000  -0.3210050000  -0.3661931000
 K024   K  -0.3424680000  0.3449070000  -0.4090827250
```

$(Co_{0.5}Ni_{0.5}/K)_6$ CIF file

```
_cell_length_a                          2.92661445
_cell_length_b                          5.25488245
_cell_length_c                          40.00000000
_cell_angle_alpha                       90.000000
_cell_angle_beta                        90.000000
_cell_angle_gamma                       90.000000
_symmetry_space_group_name_H-M          'P 1'
_symmetry_Int_Tables_number             1

loop_
_symmetry_equiv_pos_as_xyz
   'x, y, z'

loop_
   _atom_site_label
   _atom_site_type_symbol
   _atom_site_fract_x
   _atom_site_fract_y
   _atom_site_fract_z
 K001   K  -0.5000000000  -0.0197543959  0.4094627858
 K002   K  0.0000000000  0.4769053366  0.4047455755
 O003   O  -0.5000000000  -0.3306318041  0.3653152136
 O004   O  0.0000000000  0.1343295191  0.3652307843
NI005  NI  -0.5000000000  0.3137064655  0.3383383045
CO006  CO  0.0000000000  -0.1830610813  0.3391014424
 O007   O  0.0000000000  0.4972334192  0.3150986328
 O008   O  -0.5000000000  -0.0347980723  0.3144966177
 K009   K  -0.5000000000  -0.3471390210  0.2656592720
 K010   K  0.0000000000  0.1512592557  0.2669321487
 O011   O  -0.5000000000  0.3257968264  0.2191478314
 O012   O  0.0000000000  -0.1917923569  0.2164755458
NI013  NI  -0.5000000000  -0.0264318770  0.1936905605
CO014  CO  0.0000000000  0.4728032941  0.1937933926
 O015   O  0.0000000000  0.1393546067  0.1708789761
```

O016	O	-0.5000000000	-0.3811214564	0.1684927776
K017	K	-0.5000000000	0.2963720563	0.1212524055
K018	K	0.0000000000	-0.2037747526	0.1213325550
O019	O	-0.5000000000	-0.0269212665	0.0741246604
O020	O	0.0000000000	0.4535804628	0.0716656271
NI021	NI	-0.5000000000	-0.3805675084	0.0488309751
CO022	CO	0.0000000000	0.1193838570	0.0488348863
O023	O	0.0000000000	-0.2147095508	0.0260096951
O024	O	-0.5000000000	0.2656960495	0.0235612057
K025	K	-0.5000000000	-0.0574467053	-0.0235910832
K026	K	-0.0000000000	0.4425532947	-0.0235910832
O027	O	-0.5000000000	-0.3806701159	-0.0707732497
O028	O	-0.0000000000	0.0997721280	-0.0732157571
NI029	NI	-0.5000000000	0.2659572132	-0.0961120891
CO030	CO	-0.0000000000	-0.2340427868	-0.0961120891
O031	O	-0.0000000000	0.4321422984	-0.1190084211
O032	O	-0.5000000000	-0.0874154577	-0.1214509286
K033	K	-0.5000000000	-0.4106388683	-0.1686330950
K034	K	-0.0000000000	0.0893611317	-0.1686330950
O035	O	-0.5000000000	0.2661377211	-0.2158152614
O036	O	-0.0000000000	-0.2534200349	-0.2182577689
NI037	NI	-0.5000000000	-0.0872349498	-0.2411541009
CO038	CO	-0.0000000000	0.4127650502	-0.2411541009
O039	O	-0.0000000000	0.0789501354	-0.2640504329
O040	O	-0.5000000000	-0.4406076207	-0.2664929404
K041	K	-0.5000000000	0.2361689687	-0.3136751068
K042	K	-0.0000000000	-0.2638310313	-0.3136751068
O043	O	-0.5000000000	-0.0870544419	-0.3608572732
O044	O	-0.0000000000	0.3933878021	-0.3632997807
NI045	NI	-0.5000000000	-0.4404271128	-0.3861961127
CO046	CO	-0.0000000000	0.0595728872	-0.3861961127
O047	O	-0.0000000000	-0.2742420276	-0.4090924447
O048	O	-0.5000000000	0.2062002164	-0.4115349521
